# Modeling of the axon membrane skeleton structure and implications for its mechanical properties

**DOI:** 10.1371/journal.pcbi.1005407

**Published:** 2017-02-27

**Authors:** Yihao Zhang, Krithika Abiraman, He Li, David M. Pierce, Anastasios V. Tzingounis, George Lykotrafitis

**Affiliations:** 1 Department of Mechanical Engineering, University of Connecticut, Storrs, Connecticut, United States of America; 2 Department of Biomedical Engineering, University of Connecticut, Storrs, Connecticut, United States of America; 3 Division of Applied Mathematics, Brown University, Providence, Rhode Island, United States of America; 4 Department of Mathematics, University of Connecticut, Storrs, Connecticut, United States of America; 5 Department of Physiology and Neurobiology, University of Connecticut, Storrs, Connecticut, United States of America; Università della Svizzera Italiana, SWITZERLAND

## Abstract

Super-resolution microscopy recently revealed that, unlike the soma and dendrites, the axon membrane skeleton is structured as a series of actin rings connected by spectrin filaments that are held under tension. Currently, the structure-function relationship of the axonal structure is unclear. Here, we used atomic force microscopy (AFM) to show that the stiffness of the axon plasma membrane is significantly higher than the stiffnesses of dendrites and somata. To examine whether the structure of the axon plasma membrane determines its overall stiffness, we introduced a coarse-grain molecular dynamics model of the axon membrane skeleton that reproduces the structure identified by super-resolution microscopy. Our proposed computational model accurately simulates the median value of the Young’s modulus of the axon plasma membrane determined by atomic force microscopy. It also predicts that because the spectrin filaments are under entropic tension, the thermal random motion of the voltage-gated sodium channels (Na_v_), which are bound to ankyrin particles, a critical axonal protein, is reduced compared to the thermal motion when spectrin filaments are held at equilibrium. Lastly, our model predicts that because spectrin filaments are under tension, any axonal injuries that lacerate spectrin filaments will likely lead to a permanent disruption of the membrane skeleton due to the inability of spectrin filaments to spontaneously form their initial under-tension configuration.

## Author summary

Super-resolution microscopy has suggested that the actin cytoskeleton structure differ between various neuronal subcompartments. To determine the possible implication of the differing actin cytoskeleton structure, we determined the stiffness of the plasma membrane of neuronal subcompartments using atomic force microscopy (AFM). We found that axons are almost ~6 fold stiffer than the soma and ~2 fold stiffer than dendrites. By using a particle-based model for the surface membrane skeleton of the axon that comprises actin rings connected with spring filaments to represent the axonal structure, we show that regions neighboring actin rings are stiffer than areas between these rings. In these in between sub-regions, the spectrin filaments determine stiffness. Our modeling also shows that because the spectrin filaments are under tension, the thermal jitter of the actin-associated ankyrin particles, connected to the middle area of spectrin filaments, is minimal. As a result, we propose that the sodium channels bound to ankyrin particles will maintain an ordered distribution along the axon. We also predict that laceration of the spectrin filaments due to injury will cause a permanent damage to the axon since spontaneous repair of the spectrin network is not possible as spectrin filaments are under entropic tension.

## Introduction

**Fig 1:**
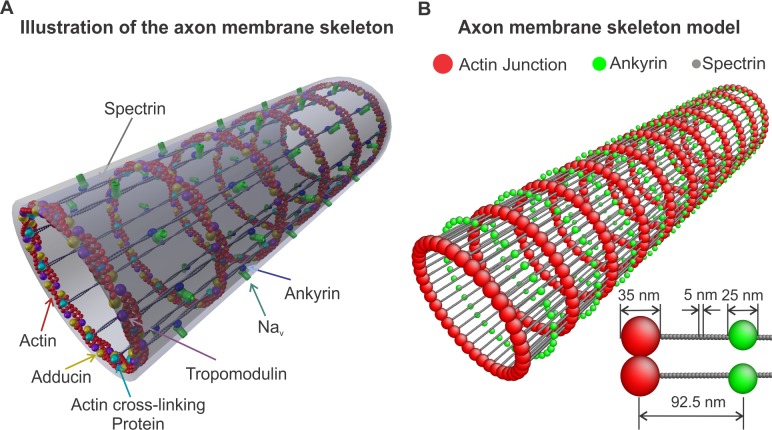
Axon membrane skeleton model. (A) Illustration of the axon membrane skeleton based on super-resolution microscopy results [3] exhibiting actin rings connected by spectrin tetramers. Ankyrin associated Na_v_ channels anchor the lipid bilayer to the membrane skeleton. Adducin has also been observed to colocalize with the actin rings possibly capping actin filaments. (B) A coarse-grain membrane skeleton dynamics model comprising representation of actin rings, spectrin filaments, and ankyrin. The insert shows the dimensions of the considered particles. (S2 Fig)

It is known for some time that microtubules and neurofilaments are the predominant structural filamentous proteins in the axon [1, 2]. However, how these filaments are arranged to generate the structure of the axon plasma membrane skeleton was only very recently discovered [3]. Super-resolution fluorescence microscopy [4] revealed that the membrane skeleton of an unmyelinated axon consists of actin filaments, capped with adducin at one end, that form ring-like structures along the circumference of the axon. The actin rings are connected via spectrin tetramers oriented along the longitudinal direction of the axon (Fig 1A). This cytoskeletal structure is extended across the entire axon. The distance between the periodic actin rings is approximately 180 to 190 nm [3, 5, 6]. Each spectrin tetramer is formed by the association of two identical heterodimers comprising an α-chain and a β-chain with 22 and 17-triple-helical segments, respectively [7]. In the distal axon, each heterodimer consists of two intertwined αII- and βII-spectrin chains running antiparallel to one another (Figure A in S1 Fig). The axon initial segment (AIS) of mature neurons, however, appears to contain the subtype βIV-spectrin instead of βII-spectrin [5].

Spectrin tetramers are associated with the lipid bilayer [3] in a manner similar to that occurring at the red blood cell (RBC) membrane [7, 8] (Figure B in S1 Fig). In RBCs, ankyrin plays a major role in anchoring the lipid bilayer to the spectrin network by associating a spectrin filament with the anion exchanger integral membrane protein band-3 [9, 10] (Figure A in S1 Fig). Ankyrin binds to the middle of the spectrin tetramer, at the 15^th^ repeat of β-spectrin near its carboxyl terminus [7, 11], and at the same time it binds to the cytoplasmic domain of band-3 [9]. In axons, the spatial distribution of ankyrin-G and ankyrin-B is highly periodic in the proximal and distal area of the axon, respectively [3, 5]. Ankyrin, besides anchoring the spectrin network to the lipid bilayer, is critical for the organization of the axonal plasma membrane because it binds to several molecules. Voltage-gated Na (Na_v_) channels can bind to subdomains 3 and 4 of ankyrin [12]. Since ankyrin has a periodic pattern in the axon, the Na_v_ channels also exhibit a periodic distribution pattern in the AIS alternating with the N terminus of βIV-spectrin [3].

The periodic structure of the membrane skeleton is thought to play an important role in the structural durability of the axon [3]. This conjecture comes from the similarity between the structural elements of the plasma membrane skeletons of the RBC and of the neuronal axon. However, we note that their geometrical arrangements are radically different resulting in hyperelastic flexibility for the RBC cytoskeleton and in reduced radial deformability and longitudinal extensibility for the axon. The two most important differences between the geometric configurations of the RBC and of the axon membrane skeletons are the following: first, the RBC cytoskeleton forms an approximately six-fold symmetric two-dimensional network (Figure B in S1 Fig) that behaves as an incompressible hyperelastic material [13, 14]. In the axon, actin filaments form rings along the circumference, connected by spectrin filaments oriented along the axon. In this case, the membrane skeleton assumes the form of a two-dimensional cylindrically symmetric orthotropic network (Fig 1). The second important difference is that in the case of the RBC membrane, the end-to-end distance of the spectrin tetramers is ~ 75*nm*, which is close to the end-to-end distance of a free spectrin filament [15]. This suggests that the spectrin network in the RBC is near equilibrium. In the axon, however, the distance between the actin rings was reported to be approximately 180 to 190*nm* [3, 6]. It is thought that microtubules stabilize the axon through interactions with neurofilaments, organelles [1, 2, 16] and possibly directly or indirectly with the actin rings, holding them apart at a specific distance. Since we do not know the exact configuration of actin rings, we assume that the upper limit of a junction of actin filaments and proteins promoting actin-spectrin binding is approximately 35 *nm*, which is close to the size of actin junctions in RBCs [17]. Thus, we consider that the end-to-end distance of the spectrin tetramers is approximately 150 *nm* while their contour length is approximately 200 *nm* [18, 19]. This means that the spectrin filaments in the axon membrane skeleton are held under entropic tension suggesting that the flexibility of the network along the axon might be limited. However, because we do not know the exact thickness of the actin rings and consequently the end-to-end distance of the spectrin filaments, we also considered cases where the size of the junctions between actin and spectrin filaments were 25*nm*, 15*nm*, and 5*nm*. The diameter of an actin filament is approximately 8*nm* [20, 21].

Based on the particular structure of the axon, we expect that its mechanical properties are different than the mechanical properties of soma and dendrites. Here, we used atomic force microscopy (AFM) to measure, via indentation, the stiffness of the plasma membrane of the subcompartments of cultured hippocampal pyramidal neurons. Importantly, we developed a coarse-grain molecular dynamics (CGMD) model for the membrane skeleton of the axon that comprises representation and connectivities of its main filaments. Based on the AFM measurements and on geometric and material parameters for the implemented filament models available in literature, we were able to examine the effect of the particular geometric configuration of the membrane skeleton and reproduce the stiffness of the axon plasma membrane. We note that while the model represents only the axon plasma membrane and considers all connectivities between the different components mostly as stable and not as dynamic processes, it provides a clear picture of how spectrin filaments in conjunction with actin rings contribute to the mechanical properties of the axonal membrane. We expect that the model will be used in studies of the mechanical stability of the axon, and generation and propagation of the action potential.

## Materials and models

### Primary culture of rat hippocampal neurons

E18 hippocampal tissue obtained from BrainBits (BrainBits, Springfield, IL) was treated with trypsin and plated onto poly-D-lysine (Sigma Aldrich, St. Louis, MO)-coated glass bottom petri dishes (Ted Pella, Redding, CA) in neurobasal media (Thermo Scientific, Waltham, MA) supplemented with B27 (Thermo Scientific, Waltham, MA), penicillin streptomycin (Thermo Scientific, Waltham, MA) and glutamax (Thermo Scientific, Waltham, MA). The cells were maintained at 37°C in a humidified incubator with 5% CO_2_ until use.

### Identification of axon and dendrites

After 8–10 DIV, neurons were transfected with tau-gfp using Lipofectamine 2000 according to manufacturer directions (Thermo Scientific, Waltham, MA). Tau-gfp was used to visualize axons in living neurons. Although tau-gfp tagged both axons and dendrites, axons were identified by their distinct morphology (S3 Fig). Tau-gfp was a gift from Dr. Walikonis, Department of Physiology and Neurobiology, UCONN, Storrs.

### Finite element model for the indentation of a thin-walled cylinder of a neo-Hookean material using a conical indenter with a spherical tip

We obtained the Young’s moduli of the axon plasma membrane, dendrites, and soma via AFM indentation experiments with a maximum displacement of 200 *nm*. In these cases, the simple Hertz contact model of elastic half-space indentation cannot be used because cells do not behave elastically under large deformations and because of the geometric characteristics of dendrites and axon. Instead, we implemented a finite element model (FEM) to compute force-indentation (*F* − *d*) relationships and used them to obtain the Young’s moduli for soma, dendrites, and axon. The method and results are explained in detail in the S1 Text. Below, we briefly describe the FE approach.

Large deformations of cell plasma membranes can be described reasonably well using the nearly incompressible neo-Hookean constitutive model [13, 22, 23]. We employ the isochoric deformation gradient $\bar{F}=J^{-1/3}F$, and similarly the isochoric right Cauchy-Green tensor $\bar{C}=J^{-2/3}C$, where **C** = **F**^T^**F** and *J* is the Jacobian, the determinant of the deformation gradient **F**. For the neo-Hookean model $\psi =\frac{1}{c}{\left(J-1\right)}^{2}+\frac{\mu }{2}\left({\bar{I}}_{1}-3\right)$, where *c* = 6(1−2*ν*)/*E* = 2/*κ*, *E* is the initial Young’s modulus, *ν* is the Poisson’s ratio, and *κ* is the initial bulk modulus of the material. In the case of incompressibility, *c* degenerates to a nonphysical, positive penalty parameter used to enforce incompressibility. The parameter *μ* is the initial shear modulus, and ${\bar{I}}_{1}=\text{tr}\bar{C}$ is the first invariant of the isochoric right Cauchy-Green tensor. From the deformation gradient, we calculate Cauchy stress **σ** as $\sigma =\frac{2}{J}F\frac{\partial \psi }{\partial C}F^{\text{T}}$. Applying the nearly incompressible neo-Hookean material model, we simulate indentation of both (*i*) a homogeneous isotropic rectangular cuboid and (*ii*) a homogeneous and isotropic thin-walled cylinder, by a conical indenter with a blunt tip using FE analyses in ANSYS workbench 14.0 (Canonsburg, PA). We note that in the actual AFM experiments, the cantilever tip was of a pyramidal shape while in the FEM calculations we used a conical indenter with a blunt tip to avoid complications stemming from the pyramid edges. However, we show in the S1 Text that the *F* − *d* relationship valid for a pyramidal indenter, with a blunt tip with a semi-included angle of 20° and a tip radius of 20 *nm*, is very similar to the *F* − *d* relationship for a conical indenter with the same semi-included angle and tip radius (S4 Fig). Because of this, we expect that the FEM results are suitable in the calculation of the Young’s moduli from the AFM indentation experiments as we explain in the section below.

### Measurements of axon plasma membrane stiffness

We carried out stiffness measurements on living rat hippocampal pyramidal neurons (16–18 DIV) using AFM silicon nitride cantilevers with a nominal spring constant of 0.03 N/m (MLCT, Bruker Probes, Camarillo, CA). Exact values for the cantilever spring constants were obtained via a thermal noise based method implemented by the manufacturer and were used in all calculations. Probes were of four-sided pyramidal shape with nominal tip radius of 20 *nm* and nominal semi-included angle of approximately 20°, as provided by the manufacturer. The tip was indented ~ 200 *nm* into the cell. Only ~ 100 *nm* of this indentation was used to determine the Young's modulus. The diameter of the axon at the measurement locations was approximately 1 *μm*. We note that in pyramidal neurons, microtubules, and neurofilaments are located at a distance greater than 200 *nm* from the axonal membrane [24, 25]. Because the indentation depth in our experiments was approximately 100 *nm*, we do not expect that microtubules and neurofilaments will contribute to the measured axon plasma membrane stiffness. The same argument is true for dendrites since microtubules are located at more than 200 *nm* distance from dendritic plasma membrane [26]. For dendrite stiffness measurements, we tested areas where the dendrite diameter was larger than 2 *μm*. Measurements of soma stiffness were performed at different regions of the soma excluding the area over the nucleus. All measurements were performed in supplemented neurobasal media at 37°*C*.

To measure the axon plasma membrane stiffness, we performed indentations at 16 x 16 points distributed uniformly in a 500 *nm*×500 *nm* area of the axon surface at a loading rate of 10,000 *pN*/*s*. For each measurement the cantilever displacement was calibrated at the rigid substrate next to the cell. Young's moduli *E* were calculated based on the force-indentation (*F* − *d*) relationship (Eq S7) for a conical indenter with a blunt tip, with a semi-included angle *θ* = 20° and a tip radius of *R* = 20 *nm*, indenting (up to 200 *nm*) a neo-Hookean thin-walled cylinder of 1 *μm* diameter and of *h* = 10 *nm* wall-thickness. For the assessment of the plasma membrane stiffness of dendrites we followed the same approach as with the axon plasma membrane and used the same equation (Eq S7). In addition, the Young’s modulus of soma was calculated based on the (*F* − *d*) relationship (Eq S5). The soma was simulated as a nearly incompressible (Poisson’s ratio *ν* ≃ 0.5) neo-Hookean rectangular 10 *μm*×10 *μm*×5 *μm* homogeneous and isotropic cuboid. In the S1 Text we show that the (*F* − *d*) for the thin-walled cylinder is *F* = (4.41×10^−3^ *E*)*d*^1.37^ (Eq S7), and for a rectangular cuboid is *F* = (7.95×10^−3^ *E*)*d*^1.46^ (Eq S5). We note that the above (*F* − *d*) relationships are valid when the indentation *d* is measured in *nm*, the initial Young’s modulus *E* in *kPa*, and the force *F* in *pN*.

### Data processing and results

**Fig 2:**
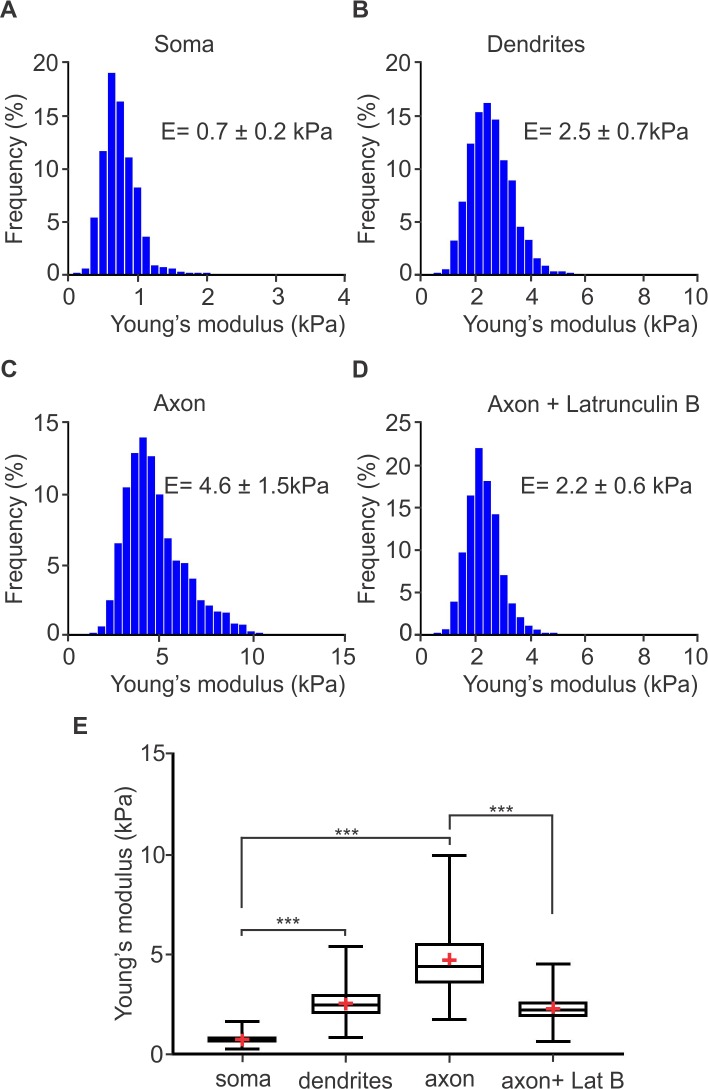
Young's moduli of rat hippocampal neuronal subcompartments determined by AFM. Histograms of Young’s moduli of rat hippocampal (A) soma, (B) dendrites, (C) axons, and (D) axons treated with 20μm Latrunculin B. The median Young's modulus of the soma is 0.7 ± 0.2 *kPa* (A), of dendrites is 2.5 ± 0.7 *kPa* (B). For the axon plasma membrane, the median Young’s modulus is 4.6 ± 1.5 *kPa* (C). When axons were treated with Latrunculin B (20μm, 1 hour) the median value of the axon plasma membrane Young’s modulus was reduced to 2.2 ± 0.6 *kPa*. Number of samples (N = 2), total number of tested neurons (n = 8). N = 1 and n = 6 for axon + Latrunculin B. (E) Box-whisker plots of mean Young's moduli of the soma, dendrites, axon, and axon treated with Latrunculin B. *** indicates statistical significance of *p* < 0.001 (Kruskal-Wallis test).

We used an open source program called force review automation environment (FRAME) [27], developed by our lab, to determine the Young's moduli of the soma, dendrites and axon of hippocampal neurons. A value of the Young's modulus from each force-displacement curve was determined by fitting the theoretical curve for a pyramidal indenter with a blunt tip mentioned above. The resulting stiffness for the specific neuronal sub-compartment was determined as the median value of the probability distribution plot generated by the individual measurements of stiffness at each point. We tested n neurons from N samples. The median values were determined horizontally across all samples. We found that the soma has a Young's modulus of 0.7 ± 0.2 *kPa* (N = 2, n = 8) (Fig 2A). The term frequency in Fig 2 corresponds to the percentage of measurements that gave a value within the corresponding bin range. The Young's modulus of dendrites was found to be 2.5 ± 0.7 *kPa* (N = 2, n = 8) (Fig 2B). Importantly, the axon was the stiffest sub-compartment of the neuron exhibiting a median value of the Young’s modulus of 4.6 ± 1.5 *kPa* (N = 2, n = 8). To evaluate if actin is critical for the observed high stiffness of the axon plasma membrane, we incubated neurons with Latrunculin B (20 μM for 1 hour), a compound that inhibits actin polymerization. This in turn, results in disruption of actin filaments. We found that in the presence of Latrunculin B the median value of the Young’s modulus was reduced to 2.2 ± 0.6 *kPa (N = 1, n = 6)* indicating that actin rings play a very significant role in determining the overall axon plasma membrane stiffness.

We note that our results on the Young's modulus of soma and dendrites of rat hippocampal neurons are in agreement with published results [28, 29]. However, to our knowledge, there are no published measurements of the stiffness of pyramidal neuron axon plasma membrane. It is clear that the stiffness of the axon is much higher than the stiffness of the soma and the stiffness of the dendrites. Below, we introduce a CGMD model for the axon and we then use it to understand how the axon plasma membrane skeleton structure leads to its elevated stiffness.

### Axon membrane skeleton model

The proposed model reflects the structure of the membrane skeleton of the proximal and distal unmyelinated axon as previously described [3, 5]. The model is a representation of actin rings, oriented along the circumference of the axon, that are connected by spectrin tetramers tethered to the lipid bilayer at their middle section (Fig 1).

### Modeling spectrin tetramers

A spectrin tetramer consists of two identical, intertwined, head-to-head associated heterodimers [18]. Each heterodimer is comprised of an α-spectrin and a β-spectrin chain consisting of 22 and 19 homologous triple helical repeats, respectively [7]. In our model, a spectrin tetramer is represented as a single chain of 41 spherical beads (gray particles in Figure A in S2 Fig) connected by 40 harmonic springs. The solid red line in Figure B in S2 Fig reflects the spring potential, $U^{SS}\left(r\right)=1/2k_{0}{\left(r\text{-}r_{eq}^{SS}\right)}^{2}$, where *r* is the distance between two consecutive spectrin particles, $r_{eq}^{SS}=L_{c}/40=5nm$ is the equilibrium distance between the spectrin particles (close to the size of the spectrin repeats), *L*_*c*_ ≃ 200*nm* is the contour length of the spectrin tetramers, and *k*_0_ is the spring constant (defined below). All spectrin particles interact via the repulsive Lennard-Jones (L-J) potential:

$$U_{rep}^{SS}(r_{\text{ij}})=\{\begin{array}{cc} 4\varepsilon_{1}\left[{\left(\frac{\sigma }{r_{\text{ij}}}\right)}^{12}-{\left(\frac{\sigma }{r_{\text{ij}}}\right)}^{6}\right]+\varepsilon_{1} & r_{\text{ij}}<R_{\text{cut},\text{LJ}}=r_{eq}^{SS}\\ 0 & r_{\text{ij}}>R_{\text{cut},\text{LJ}}=r_{eq}^{SS} \end{array},$$ (1)

where *ε*_1_ = (1/16)*ε* with *ε* being the energy unit, *σ* the length unit, and *r*_*ij*_ the distance between spectrin particles. The value *ε*_1_ = (1/16)*ε* gives a curvature at the equilibrium equal to the spring constant *k*_0_. Setting the diameter of the spectrin particles equal to the equilibrium distance of the L-J potential $r_{eq}^{SS}=2^{1/6}\sigma =5nm$, the length scale is *σ* = 4.45 *nm*. We discuss the energy scale in the section below related to modeling the plasma membrane network. We chose the cutoff distance of the potential *R*_*cut*,*LJ*_ to be the equilibrium distance $r_{eq}^{SS}$ between two spectrin particles. The potential is plotted as a dashed red line in Figure B in S2 Fig. We note that in order to reduce the number of free parameters of the model the spring constant was set to *k*_0_ = 3.56 *ε*/*σ*^2^ which is identical to the curvature at equilibrium of the L-J potential used in actin-spectrin interaction (see the section on Modeling the axon plasma membrane network).

We computed the end-to-end distance ${\langle r_{ee}^{2}\rangle }^{1/2}$ of the spectrin chain model for *K*_*B*_*T*/*ε* = 0.03, where *K*_*B*_ is the Boltzmann’s constant. We first equilibrated the filament for 10^5^ time steps, and then measured the end-to-end distance for 3×10^6^ time steps during its thermal fluctuations. The recorded distances follow a Gaussian distribution $P\left(r_{ee}\right)=1/\left(\lambda \sqrt{2\pi }\right)\exp\left[-{\left(r_{ee}-\langle r_{ee}\rangle \right)}^{2}/2\lambda^{2}\right]$, where $\lambda =\sqrt{\langle {\left(r_{ee}-\langle r_{ee}\rangle \right)}^{2}\rangle }$, and with a mean value of ${\langle r_{ee}^{2}\rangle }^{1/2}=74.3\;nm$ (S14 Fig). For flexible filaments with *l*_*p*_ << *L*_*c*_, the end-to-end distance is correlated with persistence length and contour length via the expression ${\langle r_{ee}^{2}\rangle }^{1/2}≅\sqrt{2l_{p}L_{c}}$. Taking into consideration that the spectrin contour length is approximately 200 *nm* [18, 19], we calculated the persistence length to be 13.8*nm*. This result is close to experimentally reported values of approximately 20 *nm* [30] and 10 *nm* [31].

### Modeling actin rings along the axon

The actin rings consist of short actin filaments arranged along the circumference of the axon [3]. The exact configuration of the aligned actin filaments and how they are connected to form the actin rings is not known. It has however been shown that adducin is present in the actin rings [3] probably capping and stabilizing the plus end of actin filaments. We then expect that the minus end of F-actin is stabilized by another protein, probably tropomodulin [21], while additional cross-linking proteins are possibly involved in the formation of the actin rings [21, 32, 33] (Fig 1A). Because the exact molecular structure of the actin rings and whether actin filaments are connected in a side-by-side or an end-to-end arrangement is not known, we adopted a coarse-grain particle model that produces stable actin rings but ignores their specific molecular structure.

In this particle model, an actin ring is represented as a collection of 39 beads (red particles in Fig 1B and insert and in Figure A in S2 Fig) with a diameter of approximately 35 *nm*. These beads form a circle with a diameter of approximately 434 *nm*, which lies within the range of experimental results [3, 5, 34]. We chose the diameter of the actin particles to be 35 *nm* based on values for the RBC membrane skeleton, which comprises short actin oligomers (consisting of approximately 13 to 15 subunits) with a length of 33 ± 5 *nm* [17, 35, 36].

Two adjacent actin particles in the same ring connect via a spring potential $U^{AA}=1/2k_{A}{\left(r-r_{eq}^{AA}\right)}^{2}$, with equilibrium distance $r_{eq}^{AA}=35nm$, and a repulsive L-J potential $U_{rep}^{AA}$ (S1 Table), with $R_{cut,LJ}=r_{eq}^{AA}$ (shown as purple lines in Figure B in S2 Fig). The value of the spring constant *k*_*A*_ = 38.0 *ε*/*σ*^2^ is determined in computational results in conjunction with the AFM stiffness measurement of the axon plasma membrane. In addition, we employed a finitely deformable nonlinear bending potential that behaves as a finitely extendable nonlinear elastic (FENE) potential to maintain the circular shape of the actin rings. The potential has the form $U_{b}=-\frac{1}{2}k_{b}\;\Delta \theta_{max}\;ln\left[1-{\left(\frac{\theta -\theta_{0}}{\Delta \theta_{max}}\right)}^{2}\right]$, where *k*_*b*_ = 3,500 *K*_*B*_*T* is the parameter that directly regulates the bending stiffness of the actin filament and it is determined in S1 Text. *θ* is the angle formed by three consecutive particles of the same ring. $\theta_{0}=\frac{180{}^{\circ}\left(39-2\right)}{39}=170.77{}^{\circ}$ is the equilibrium angle and Δ*θ*_*max*_ = 0.3*θ*_0_ is the maximum allowed bending angle. We note that the resistance of the actin ring to small deformations depends on *k*_*b*_/Δ*θ*_*max*_ since for small deformations the FENE potential is approximated by $U_{b}=\frac{1}{2}\left(k_{b}/\Delta \theta_{max}\right){\left(\theta -\theta_{0}\right)}^{2}$, which corresponds to a harmonic potential with a spring constant *k*_*bs*_ = *k*_*b*_/Δ*θ*_*max*_. This means that the exact value of Δ*θ*_*max*_ does not uniquely determines the stiffness of the structure close to equilibrium but in combination with *k*_*b*_. Δ*θ*_*max*_ defines the maximum deformation of the actin rings but its exact value does not affect the behavior of the system near equilibrium. The employed value of *k*_*b*_ produces a bending rigidity of *κ*_*bend*_ = 7.1 × 10^−26^ *Nm*^2^ for a straight stiff filament based on numerical calculations shown in S1 Text and in [37]. The obtained value is similar to the experimentally measured bending rigidity of actin filaments 7.3 × 10^−26^ *Nm*^2^ reported in [38, 39].

### Modeling the axon plasma membrane network

To build a mechanically stable network, we first connected each spectrin filament at its two ends to actin particles belonging to consecutive actin rings via a breakable L-J potential $U_{LJ}^{AS}(r_{ij})=4\varepsilon \left[{\left(4\sigma /r_{ij}\right)}^{12}-{\left(4\sigma /r_{ij}\right)}^{6}\right]$, where *r*_*ij*_ is the distance between actin and spectrin particles (blue dashed line in Figure B in S2 Fig). The equilibrium distance between actin and spectrin is 2^1/6^ (4*σ*) ≃ 20 *nm* resulting to an actin junction size of approximately 40 *nm* [40]. This equilibrium distance corresponds to an end-to-end distance of 145 *nm* for the spectrin filaments. However, because the exact thickness of the actin rings and consequently the equilibrium distance between actin and spectrin particles are not known, we also considered the cases where $r_{eq}^{AS}=15nm,\;10nm,\;\text{and}\;5nm$ (approximately the diameter of a G-actin monomer [20]), which correspond to end-to-end distance of *r*_*ee*_ = 155*nm*, 165*nm*, and 175*nm* respectively for the spectrin filaments.

We note that in this model the actin-spectrin association can break and reform. The association breaks, by setting the attractive force to zero, when the distance between the particles crosses the inflexion point of the L-J potential at *r*_*inflexion*_ = (26/7)^1/6^ 4*σ*, indicated by the red circle (blue dashed line in Figure B in S2 Fig). It can reform as the distance between actin and spectrin particles becomes smaller than the capture distance of 2.5×4*σ*. For a stable membrane skeleton, the spectrin-actin junction association energy was chosen to be *ε* ≃ *k*_*B*_*T*/0.03 ≃ 0.86 *eV* for *T* = 300°*K*. Equilibrium measurements have shown that the association energy for the spectrin-actin-protein 4.1 complex in normal RBCs is about 17 *Kcal*/*mole* = 0.74*eV* [41]. However, for this value the membrane skeleton would be partially broken when the distance between actin rings is set equal to the experimental value of 185 *nm*. This means that the association energy of the spectrin-actin complex in the axon is most likely larger than in normal RBCs, to maintain stable membrane skeleton with spectrin filaments under tension.

Microtubules and neurofilaments are thought to play an important role in maintaining the polarity and structure of the AIS through interactions with the axonal cytoskeleton. Pyramidal neurons, which we used to experimentally determine the axonal membrane stiffness, have microtubules that are not structured in bundles but rather like a string of beads (in cross-section) [24, 25, 42] and they are at a distance greater than 200 nm from the axonal membrane [25]. Similarly, neurofilaments in pyramidal neurons are not arranged in bundles and are much farther away from the axonal membrane than microtubules [24]. Since the indentation depth of the AFM probe was ~ 100–150nm, microtubules and neurofilaments are not expected to contribute to axonal membrane stiffness in our measurements. In our model, the effect of microtubules and neurofilaments on the structural integrity of the axon was implemented implicitly. We considered that microtubules interact with actin to maintain the equilibrium distance of actin rings at 185 *nm*. To achieve this, we applied the FENE potential $U_{mt}=-\frac{1}{2}\;k_{mt}\;\Delta d_{max}\;ln\left[1-{\left(\frac{d-d_{eq}^{RR}}{\Delta d_{max}}\right)}^{2}\right]$ on all actin particles belonging to consecutive rings. *k*_*mt*_ is the parameter that determines the stiffness of the nonlinear spring between two actin rings, *d* and $d_{eq}^{RR}=185nm$ are the distance and the equilibrium distance between the centers of the two actin rings, respectively [3, 5], and $\Delta d_{max}=0.3\;d_{eq}^{RR}$ is the maximum allowed deformation. The position of the center of each ring is calculated by utilizing the mean value of the *z*–coordinate of the actin particles. The choice of *k*_*mt*_ is justified based on the following rationale: for small deformations, the FENE potential is approximated by $U_{mt}=\frac{1}{2}\left(k_{mt}/\Delta d_{max}\right){\left(d-d_{eq}^{RR}\right)}^{2}$, which corresponds to a harmonic potential with a spring constant *k*_*sp*_ = *k*_*mt*_/Δ*d*_*max*_ [37]. In this case, we can assume that *τ* = *E*_*L*_*h*, where *τ* is the stress, *E*_*L*_ is the longitudinal Young’s modulus of the axon, and *h* is the strain. The final equation is *F*/*A* = *E*_*L*_ (Δ*L*/*L*), where $F=k_{sp}^{t}\;\Delta L$ is the force applied on the cross-section of the axon *A* = *πR*^2^, where $k_{sp}^{t}=k_{sp}/\left(N-1\right)$ is the spring constant for the total axon, *R* = 217 *nm* is the radius of the axon, Δ*L* is the elongation of the axon, $L=\left(N-1\right)d_{eq}^{RR}$ is the length of the axis, and *N* is the number of springs. Combining the equations above, we determined that $k_{sp}=E_{L}\pi R^{2}/d_{eq}^{RR}$ and consequently *k*_*mt*_ = *k*_*sp*_ Δ*d*_max_ = 0.3*E*_*L*_*πR*^2^. A reasonable value for the axon's longitudinal Young's modulus is *E*_*L*_ ≃ 10 *kPa* [43], resulting in $k_{mt}≃477\;K_{B}T/\sigma ≃19,822\;K_{B}T/d_{eq}^{RR}$, at *T* = 300°*K*.

The final aspect of the model is the association between the axon membrane skeleton and the lipid bilayer. In RBCs, the membrane skeleton is anchored to the lipid bilayer via glycophorin at the actin junction complexes and via the integral membrane protein band-3 and ankyrin at the middle section of spectrin tetramers [9, 10] (S1 Fig). Regarding the association between a spectrin filament and the lipid bilayer in RBCs, ankyrin binds at the 15^th^ repeat of β-spectrin near its carboxyl terminus, at the middle section of the spectrin tetramer [7]. At the same time, it binds to the cytoplasmic domain of band-3 [9], mediating the anchoring of spectrin filaments to the lipid bilayer. In the case of the axon, we considered the following experimental findings: (i) The spatial distribution of ankyrin-G is highly periodic in the proximal area of the axon, while ankyrin-B also exhibits a periodic pattern in distal axons [3, 5], (ii) Na_v_ channels exhibit a periodic distribution pattern in the AIS alternating with actin rings [3], (iii) Na_v_ can bind to subdomains 3 and 4 of ankyrin [12], and (iv) Ankyrin-G and sodium channels are in 1:1 molar ratio in the brain. Based on these findings and on the fact that ankyrin binds near the carboxyl terminus of β-spectrin it is reasonable to assume that Na_v_ channels are arranged in a periodic pattern along the axon via their association with ankyrin in a manner similar to band-3 association with spectrin in the RBC membrane. We also note that by assigning one Na_v_ channel per ankyrin molecule, and consequently per spectrin tetramer, the Na_v_ channel density is approximately 150 channels per μm^2^, which lies within the range of 110 to 300 channels per μm^2^ in AIS [44].

To represent the anchoring of spectrin tetramers to the lipid bilayer, we used the following approach: We connected an ankyrin particle (depicted as a green particle in Fig 1B and Figure A in S2 Fig) to the 20^th^ particle of the spectrin filament by the spring potential ${\text{U}}^{SK}\left(r_{ij}\right)=1/2{\text{k}}_{0}{\left(r_{ij}-{\text{r}}_{eq}^{SK}\right)}^{2}$, where the radial equilibrium distance is $r_{eq}^{SK}=15nm$, (black solid line in Figure B in S2 Fig). This distance corresponds to the radius of a spectrin particle (2.5 *nm*) and the effective radius of the cytoplasmic domain of the ankyrin complex connected to an Na_v_ channel (~ 12.5 *nm*) [45]. We also implemented a repulsive L-J potential $U_{rep}^{SK}$ (S1 Table), with $R_{cut,LJ}=r_{eq}^{SK}$ (dashed black line in Figure B in S2 Fig) to simulate a steric repulsion between the particles that represent spectrin and ankyrin. For simplicity, we did not use a representation of the lipid bilayer in this model. Instead, we used a spring potential to represent the confinement applied on the motion of ankyrin particles and spectrin filaments by the lipid bilayer. The harmonic confining potential is given by U^*C*^ (*r*) = 1/2k_*c*_(*r* − r_0_)^2^, where *r* is the radial distance of spectrin and ankyrin particles from the central axis of the axon, and *r*_0_ is the equilibrium distance from the central axis. We considered *r*_0_ to be 217 *nm* and 232 *nm* for the spectrin and ankyrin particles, respectively. Because the ankyrin particles are attached to the bilayer, the confinement potential acts on both radial directions (inwards and outwards). However, only the outward motion of the spectrin particles is confined, since the spectrin filament cannot cross the lipid bilayer. In contrast, the inward motion will not face additional constrain. The confinement mild stiffness in this model is arbitrarily chosen to be *k*_*c*_ = 0.1*k*_0_ since it is due to the bending rigidity of the lipid bilayer which is in the range of (10–20*K*_*B*_*T*). We finally note that an important consideration in RBC membrane modeling is that mutations can cause disruption of the association between ankyrin and spectrin resulting in stiffer skeleton, local membrane instabilities, and vesiculation [10, 46–48]. Here, we opted to focus on establishing the membrane model and explore possible membrane skeleton defects and their effect on the axon plasma membrane in a later work.

### Membrane skeleton dynamics simulation details

The configuration used in this paper consists of N = 16,029 particles, corresponding to an axon length of approximately 1.85 *μm*. The numerical integrations of the equations of motion are performed using the Beeman algorithm. The temperature of the system is maintained at *K*_*B*_*T*/*ε* = 0.03 by employing the Berendsen’s thermostat [49], where K_B_ is Boltzmann’s constant and T is the temperature. The model is implemented in the *NVT* ensemble (constant number of particles *N*, constant volume *V*, and constant temperature *T*). The time scale is $t_{s}=\sqrt{m\sigma^{2}/\varepsilon }$, the time step is *dt* = 0.01*t*_*s*_, and *m* is the unit mass of the spectrin particles. We selected the temperature to render the conformation time of the spectrin filaments close to expected theoretical values [50]. We gradually brought the model to the equilibrium length and temperature, and then equilibrated it for 15×10^4^ time steps. We performed the measurements during a period of 10×10^6^ time steps after equilibration.

## Results and discussion

In this section, we compute the stiffness associated with the introduced axon model and we explore the effect of the axon structure to the thermal motion of ankyrin particles and the distribution of connected Na_v_ channels.

### Stiffness of the axon plasma membrane

**Fig 3:**
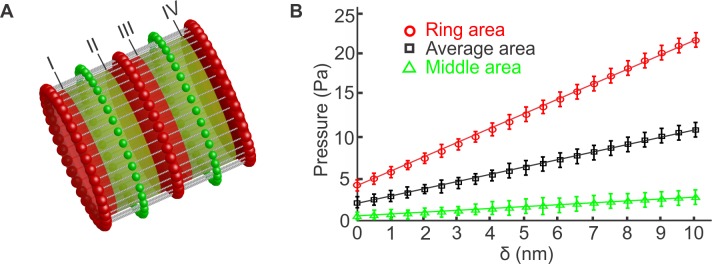
Measurement of the axon plasma membrane Young’s modulus. (A) We expand the radius of the axon by 10 *nm*, in sequential steps of 0.5 *nm*, by applying a cylindrical Lennard-Jones potential with equilibrium distance measured from the center-line of the axon. At each radius, we measure the total force applied on all the membrane skeleton particles that belong (i) to stripes of 80 *nm* diameter around the actin rings (red stripes), (ii) to stripes of 105 *nm* diameter located between actin rings (green stripes), and (iii) to the entire membrane skeleton along the axon. By dividing the total force with the corresponding total area, we compute the pressure on the region between actin rings, on the region around the actin rings and the average pressure on the entire membrane skeleton. Every 0.5 *nm* increase occurs in 1000 time steps. At each radius, the system is equilibrated for another 1000 time steps while the actual measurements occur every time step for 8000 time steps. Labels I-IV indicate the boundaries of the areas on which we measure the pressure. (B) The mean pressure values obtained for each stripe type are plotted vs the corresponding radii values. From the slope of the fitted straight line and using the linear elastic cylindrical shell theory, the Young’s moduli for each region and the average value are computed. The probability distributions of the pressures measured for the two stripes and for the entire membrane skeleton at 1.5 nm radius are shown in S16 Fig.

To measure the stiffness of the simulated axon plasma membrane skeleton, we defined a cylindrical Lennard-Jones repulsive potential $U_{LJ}^{S}(r_{ij})=4\varepsilon \left[{\left(\sigma /\left(r_{i}-\left(r_{c}-r_{S}\right)\right)\right)}^{12}-{\left(\sigma /\left(r_{i}-\left(r_{c}-r_{S}\right)\right)\right)}^{6}\right]$, for the spectrin particles and $U_{LJ}^{A}(r_{ij})=4\varepsilon \left[{\left(7\sigma /\left(r_{i}-\left(r_{c}-r_{A}\right)\right)\right)}^{12}-{\left(7\sigma /\left(r_{i}-\left(r_{c}-r_{A}\right)\right)\right)}^{6}\right]$, for the actin particles. *r*_*c*_ is the distance between the center-line of the axon and the surface of an imaginary cylinder (Fig 3A), *r*_*i*_ is the distance between particle *i* and the center-line of the axon, *r*_*S*_ is the radius of the spectrin particle and *r*_*A*_ is the radius of the actin particle. The cutoff distances of the L-J potentials are *r*_*c*_ + *r*_*S*_ and *r*_*c*_ + *r*_*A*_ for $U_{LJ}^{S}$ and $U_{LJ}^{A}$ respectively. Then, we gradually expanded the cylindrical potential to apply internal radial pressure to the membrane skeleton of the axon. The total increase of the radius was 10 *nm*, from 217 *nm* to 227 *nm*, which corresponds to approximately 4.6% of the initial radius of the axon. An axon, because of its structure, has two well separated areas in terms of lateral stiffness. We expect that the stiffness is lower at the middle region between the actin rings and higher at the region near the actin rings. The radius of an actin particle is *r*_*A*_ = 17.5 *nm* and the radius of a spectrin particle is *r*_*S*_ = 2.5 *nm*. The capture radius of the actin-spectrin junction is 2.5(*r*_*A*_ + *r*_*S*_)/2^1/6^ ≃ 44.5 *nm*. Based on this calculation, we chose the width of the stripes at the actin rings (red stripes) to be 80 *nm* and consequently the width of the stripes between the two actin rings (green stripes) is 105 *nm* since the distance between two consecutive actin rings is 185 *nm*. First, we computed the repulsive Lennard-Jones forces applied to all particles belonging to the green stripes located between consecutive actin rings at each expansion step. The total computed force was divided by the total area of all green stripes to estimate the applied pressure. We measured the pressure every 0.5 *nm* expansion increment. The transition from one radius, where we measured the pressure, to the next one lasted 1000 time steps. At each measurement, we first equilibrated the system for 1000 steps and then computed forces for 8000 time steps. The pressure values were plotted in a histogram that was approximated as a Gaussian distribution (S16 Fig (green)). After completion of the entire deformation, we plotted the mean values along with the standard deviations as function of the expansion (Fig 3B (green)). We found that the relation between pressure and the change in the radius was linear and we used the least square method to compute the slope of the fitted straight line. Using the linear elastic cylindrical shell theory, we correlated the slope with the corresponding Young's modulus *E* via the expression *E* = *pR*^2^ / *δH*, where *p* is the applied pressure, *R* is the radius of the shell, *H* is the thickness of the shell, and *δ* is the radius change. By using this equation and assuming that the thickness of the axonal membrane skeleton is *H* = 10 *nm*, we obtained the stiffness of the membrane at the area between the actin rings to be approximately *E* = 7.22 × 10^−4^ *ε*/*σ*^3^ which corresponds to *E* = 1.13 *kPa*. This result is lower than the experimentally measured axon plasma membrane stiffness when the axon was treated with Latrunculin B, which disrupts actin filaments (Fig 2D). We also note that our model does not have free parameters for this section of the axon since the main skeleton filaments that resist deformation are the spectrin filaments, for which the persistence length, geometric configuration, and connectivity to actin are known.

While the material parameters of the spectrin filaments and their geometric configuration are known, how the G-actin filaments are connected to form the actin rings is unknown. Here, we assume that G-actin filaments, represented by one particle, are connected to each other to form one-particle thick rings. In our model, we use a spring harmonic potential to maintain the equilibrium distance between two consecutive particles and a bending FENE potential to maintain the included angle between two consecutive bonds formed between three particles. The spring potential resists changes to the radius of the actin rings while the bending potential resists to changes of the circular shape of the actin rings. The spring constant *k*_*A*_ is determined below.

To measure the axon plasma membrane stiffness in the area near the actin rings, we repeated the same procedure which we followed to measure the stiffness in the area between the actin rings. In particular, we measured the applied pressure to stripes of 80 *nm* width located over the actin rings (red stripes in Fig 3A). After reiteration, we found that by employing a spring constant of *k*_*A*_ = 38.0 *ε*/*σ*^2^ the Young’s modulus is approximately *E* = 53 × 10^−4^ *ε*/*σ*^3^. This corresponds approximately to *E* = 8.3 *KPa*. We note that the actin spring constant corresponds to approximately *k*_*A*_ ≃ 0.26 *N*/*m* which is larger than the value used in previous actin filament simulations [33]. The difference is perhaps due to an enhanced connectivity between the actin filaments that form the actin ring. We finally note that by measuring the pressure applied to the entire axon in relation to the increase of the radius (Fig 3B, black) and then employing the linear shell theory, we determined that the average axon plasma membrane Young's modulus is approximately *E* = 27 × 10^−4^ *ε*/*σ*^3^. This corresponds approximately to *E* = 4.23 *KPa*. Therefore, we conclude that the experimentally determined *E* depends on both the actin rings and spectrin filaments, with actin sustaining almost six times the applied load compared to spectrin during volumetric expansion.

### Thermal motion of ankyrin particles

**Fig 4:**
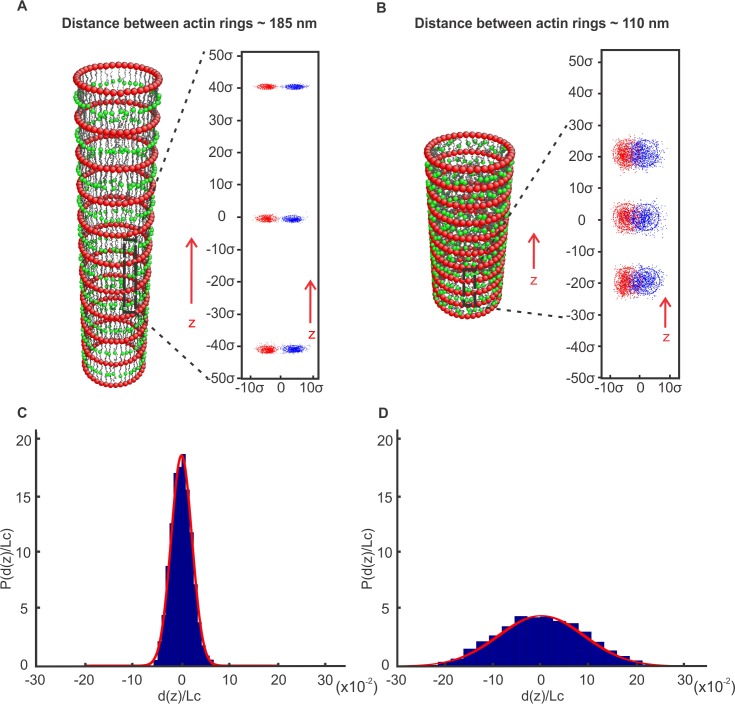
Membrane skeleton dynamics simulations. (A) The membrane skeleton was equilibrated at a distance of approximately 185 *nm* between actin rings while the trajectory of ankyrin particles (insert) was recorded for 10×10^6^ time steps every 10^5^ time steps. (B) The skeleton was equilibrated at a distance of approximately 110 *nm* between actin rings while the trajectory of ankyrin particles (insert) was recorded for 10×10^6^ 10^5^ time steps. (C, D) Normalized probability distribution of the ratio *d*(*z*)/*L*_*c*_, where *d*(*z*) is the deviation of an ankyrin point from its mean position during its thermal motion along the z-direction and *L*_*c*_ is the mean distance between two consecutive ankyrin points along the z-direction when the spectrin is under tension *L*_*c*_ = 185.78 *nm* (C) and when the spectrin is almost at equilibrium *L*_*c*_ = 112.32 *nm* (D). The longitudinal and circumferential separations of the trajectories of neighboring ankyrin particles, and consequently of the corresponding Na_v_ channels, are well-defined in (A) but not in (B).

Ankyrin proteins, depicted as green particles in Fig 1B, are connected to spectrin by a harmonic potential. Because the persistence length (*l*_*p*_) of a spectrin filament is typically considered to be between 10 *nm* and 20 *nm* [30, 31], and its contour length (*L*_*c*_) is approximately 200*nm*, we find, based on the equation ${\langle r_{ee}^{2}\rangle }^{1/2}≅\sqrt{2l_{p}L_{c}}$, that the end-to-end distance of a spectrin filament at equilibrium ranges from 63*nm* to 89.5*nm*. Spectrin tetramers of the axon membrane skeleton have an end-to-end distance of approximately 150*nm*; therefore, they are under tension with a reduced range of thermal motion. To demonstrate this, we equilibrated the model for 15×10^4^ time steps at constant volume and temperature (Fig 4A) and recorded the thermal motion of ankyrin particles for 10×10^6^ time steps once every 10^4^ time steps. We note that after 10^5^ time steps the size of the area described by the ankyrin particles hardly changes. The trajectory of ankyrin particles outlined an area with an average radius of ~ 5.37 *nm*. As Fig 4A shows, the ankyrin particles and consequently the connected Na_v_ channels maintained an ordered configuration, in contrast to simulations with spectrin not under tension (Fig 4B; equilibrium end-to-end distance of 75 *nm*). To clearly distinguish between the two cases, we plotted the distribution of the ratios *d*(*z*)/*L*_*c*_, where *d*(*z*) is the deviation of an ankyrin point from its mean position during its thermal motion along the z-direction and *L*_*c*_ is the mean distance between two consecutive ankyrin points along the z-direction when the spectrin is under tension (Fig 4C, *L*_*c*_ = 185.78 *nm*) and when the spectrin is almost at equilibrium (Fig 4D, *L*_*c*_ = 112.32 *nm*). The distribution in Fig 4D is much wider (standard deviation *s* = 0.091) than the distribution in Fig 4C (*s* = 0.027). As a consequence, when spectrin is under tension the positions of consecutive Nav channels along the axon (z-direction) are more ordered (Fig 4A and 4C) than when spectrin is at equilibrium (Fig 4B and 4D). We note that previous work has shown that increasing mobility of sodium channels in the AIS by inhibiting actin polymerization alters action potential properties [51]. Therefore, we conjecture that if the spectrin filaments were at near equilibrium, their thermal motion could affect the generation and propagation of a synchronized action potential.

We finally note that, as in the case of the RBC membrane [48, 52–55], the axonal membrane skeleton is expected to confine the lateral diffusion of channels that are not connected to the membrane cortex within the rectangular “fenced” area between two consecutive actin rings and two neighboring spectrin filaments. Because in the axon the spectrin filaments are under tension with reduced oscillation amplitudes, it is anticipated that escape of diffusing channels via “hop movements” from one compartment to another will be limited compared to the RBC membrane where spectrin filaments are not under tension and the network is not perfect.

### Laceration of spectrin filaments

Spectrin filaments in quiescent normal RBCs are dynamically connected to actin junctions. It is known that ATP-driven dissociation of spectrin filaments from actin junctions [56] results in softening of the RBC membrane [57] and allows reconfiguration of the spectrin network when a spectrin-actin association is momentarily disrupted [58]. Within this framework, we explored if re-association between spectrin filaments and actin junctions is possible in the axon membrane skeleton purely from a mechanics point of view. This is important since inability of spectrin-actin re-association would mean that laceration of the spectrin filaments due to injury will result in a permanent damage of the axon.

**Fig 5:**
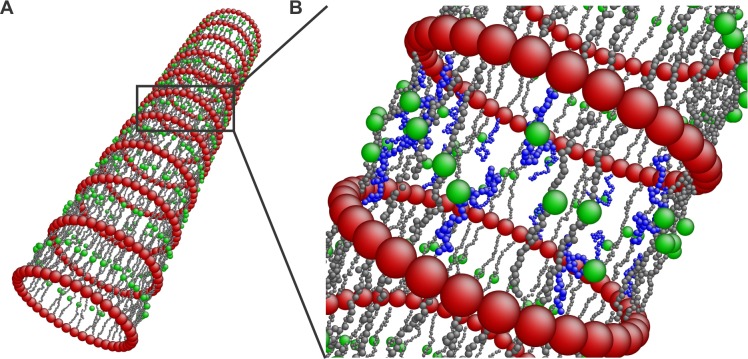
Laceration of spectrin filaments. (A) Axon membrane skeleton with severed spectrin filaments in the marked area between two consecutive rings. (B) None of the severed spectrin filaments (blue color) were reconnected to their initial junction points.

To examine this question, we considered our axon model where the distance between actin rings is 185 *nm* and 15 of the 39 spectrin filaments corresponding to each actin ring were severed. We then let the system evolve and reach equilibrium in 10^4^ time steps. After that, we allowed re-connection between severed spectrin filaments and the corresponding actin junctions for the next 10^4^ time steps. The capture radius was set at *r*_*capture*_ = 2.5×(4*σ*), equal to the cutoff distance of the L-J potential $U_{LJ}^{AS}$ between actin and spectrin particles. We observed that none of the disconnected filaments were connected back to its original junction (Fig 5). This is expected because spectrin filaments were initially under entropic tension and shrunk to their end-to-end distance at equilibrium when they were cut. However, when we considered an axon configuration with only 110 *nm* distance between actin rings, which approximately corresponds to the end-to-end distance of spectrin filaments at equilibrium, then 85% of the 15 severed filaments re-connected to their original junction in 10,000 time steps and 100% in 50,000 time steps. This result clearly demonstrates our theoretical prediction that it is very unlikely for a *normal* axon, where the distance between actin rings is approximately 185 *nm*, to recover its initial membrane skeleton configuration if spectrin filaments were at some point injured.

## Conclusion

We performed AFM experiments to measure the stiffness of the plasma membrane of the soma, dendrites and the axon of rat hippocampal pyramidal neurons. We found that the axon is much stiffer than the soma and dendrites. To understand the mechanical properties of the axon, we introduced a coarse-grain molecular dynamics model for the axon membrane skeleton of non-myelinated neurons. We found that the axon plasma membrane has two distinct Young's moduli that are correlated with its geometric structure, which is characterized by stiff actin rings connected by extended spectrin filaments oriented along the axon. We showed that the model, without using free parameters, predicts a low Young’s modulus, in the region between the actin ring. By using a spring constant for the actin filaments, similar to the one usually employed in actin filament simulations, we found a higher Young’s modulus in the region near to the actin rings. The average value of these measurements agrees with the median value of the AFM measurements of the axon plasma membrane stiffness.

In addition, we showed that because the spectrin filaments are under entropic tension, they limit the thermal motion of the attached ankyrin proteins and consequently the thermal fluctuations of the ankyrin-associated sodium channels maintaining them in a ring-like configuration. This may have an effect on the initiation and the rate-of-rise of the action potential. We also note that because spectrin filaments are under tension, axonal injuries that lacerate spectrin filaments will lead to a permanent disruption of the membrane skeleton because of the inability of spectrin filaments to spontaneously connect back to their initial, under-tension configuration.

## Supporting information

S1 Text Indentation of a neo-Hookean half space and a thin-walled cylinder using a conical indenter with a spherical tip, and measurement of the bending rigidity of actin filaments. (DOCX)

S1 Fig RBC membrane. (A) Anchoring of the lipid bilayer to the membrane skeleton. Ankyrin binds to the 15^th^ repeat of β-spectrin near its carboxyl terminus and to an anion exchanger band-3 in RBCs. α-spectrin and β-spectrin filaments are connected at actin junctions. In the axon plasma membrane, ankyrin binds to voltage-gated sodium channels (Nav). In RBCs, the NH_2_-terminal of β-spectrin binds to protein 4.1 which forms a membrane anchoring complex with glycophorin C [7]. (B) Illustration of the RBC membrane skeleton comprising stretched spectrin tetramers connected at actin junctions and exhibiting a six-fold two-dimensional symmetry. The lipid bilayer is anchored to the membrane skeleton at actin junctions by glycophorin C and near the middle of each spectrin tetramer by ankyrin which is then connected to an anion exchanger band-3 protein [7, 59].
(TIF)

S2 Fig Details of the axon membrane skeleton model. (A) Illustration of particles and connections involved in the model. Red particles represent actin junctions, gray particles represent spectrin subunits, and green particles represent ankyrin junctions. The diameter of the axon model is 434 *nm*. (B) The solid lines represent harmonic potentials applied between neighboring spectrin particles (S-S) of the same spectrin filament, spectrin and ankyrin (S-K), and between neighboring actin particles (A-A) in the actin rings. Dashed lines depict repulsive Lennard-Jones (L-J) potentials which represent steric repulsions between all particles used in the simulation. The blue dashed depicts the L-J potential applied between actin and spectrin (A-S). Note that the linkage between A-S is breakable at the inflexion distance of the potential marked with a red circle.
(TIF)

S3 Fig Fluorescent image of rat hippocampal neuron transfected with tau-gfp showing axon and AFM probe. (TIF)

S4 Fig Analytical vs simulation results for a rigid indenter with a blunt tip indenting an elastic half-space. (A) A pyramidal indenter with a blunt tip of radius 20 *nm* and a semi-included angle of 20°. (B) A conical indenter with a blunt tip of radius 20 *nm* and a semi-included angle of 20°. (C) Comparison between the analytically derived *F* − *d* curve (black solid line) for an elastic half-space indented by the conical indenter described in B and the corresponding FE (blue circles) results of indentation of an elastic 10 *μm*×10 *μm*×5 *μm* cuboid with *E* = 2 *kPa* and *ν* ≈ 0.5. The analytically derived *F* − *d* curves for the pyramidal indenter described in A and for a spherical indenter of radius 20*nm* are also shown as a solid green line and a red dashed line respectively. The *F* − *d* indentation curves for a conical and a pyramidal indenter with a blunt tip, which have the same semi-included angles and the same radii of the tips, are similar when indenting a half-space.
(TIF)

S5 Fig Finite element model of a 200 *nm* indentation of a neo-Hookean 10 *μm*×10 *μm*×5 *μm* cuboid. The color map represents the vertical displacement (z-axis) field in nm.
(TIF)

S6 Fig Analytical (linear elastic) versus simulation (neo-Hookean) results for a rigid conical indenter with a blunt tip indenting a half-space. Comparison of indentation simulation results for a neo-Hookean 10 *μm*×10 *μm*×5 *μm* cuboid of *E* = 2 *kPa* and *ν* = 0.5 with the classic Hertz solution for a rigid conical indenter with a blunt tip of 20 *nm* radius and a semi-included angle of 20° for indentations up to 200 *nm*.
(TIF)

S7 Fig Comparison of simulation results with the classic Hertz (theoretical linear elastic) solution for indentation of a neo-Hookean 1 *μm*×1 *μm*×*h* volume, where *h* is the thickness, at different thicknesses using a conical indenter ending at a spherical tip with a radius of 20 *nm* and a semi-included angle of 20° at indentations up to 50 *nm*. (TIF)

S8 Fig Indentation up to 200*nm* using a conical indenter ending at a spherical tip with a radius of 200*nm* into a nearly incompressible neo-Hookean volume 10 *μm*×10 *μm*×5 *μm* with initial Young’s modulus of *E* = 2 *kPa* and Poisson’s ratio *ν* ≈ 0.5. We assume the fitting curve is of the form *F* = *A d*^*α*^.
(TIF)

S9 Fig Relationships between (A) *E* and *A*(*E*) and (B) between *E* and *α* within a range of Elastic moduli from 0.5 *kPa* to 10 *kPa*.
(TIF)

S10 Fig Finite element analysis of a 200 *nm* indentation of a cylindrical neo-Hookean shell of 0.5 *μm* diameter and with a 10 *nm* wall-thickness. The color map represents the vertical (z-direction) displacement field measured in nm.
(TIF)

S11 Fig Force–indentation *F* − *d* dependence for a 200 *nm* indentation of a cylindrical neo-Hookean shell of 0.5 *μm* diameter and with a 10*nm* wall-thickness. (TIF)

S12 Fig Comparison of simulation results at indentations up to 200 *nm*, (A) a range of thickness from 5 *nm* to 10 *nm* with fixed *E* = 2 *kPa*, (B) a range of *E* from 2 *kPa* to 10 *kPa* with fixed thickness *h* = 10 *nm*.
(TIF)

S13 Fig Relationships (A) between *E* and *B*(*E*), and (B) between *E* and *β* determined from a range of assumed elastic moduli from 1 to 10 *kPa* for cylinder wall-thickness *h* = 10 *nm*.
(TIF)

S14 Fig Probability distribution of the recorded end-to-end distances (*r*_*ee*_) of a free spectrin filament during 3 × 10^6^ time steps of a coarse-grain solvent-free molecular dynamics simulation at constant temperature *T* = 300°*K*. The associated normalized Gaussian probability density (red line) is also shown.
(TIF)

S15 Fig Probability distribution of the recorded end displacements (*δu*) of a free actin filament during 3 × 10^6^ time steps of a coarse-grain solvent-free molecular dynamics simulation at constant temperature *T* = 300°*K*. The associated normalized Gaussian probability density is shown in red.
(TIF)

S16 Fig Probability distribution of the recorded pressures at 1.5 *nm* radius increment at the middle areas (green), ring areas (red) and at the entire axon (black). The associated normalized Gaussian probability densities are also shown.
(TIF)

S1 Table Potentials and corresponding parameters used in modeling of the axon membrane skeleton. (PDF)

S2 Table Values of *B*(×10^−3^) for Young’s modulus *E* ranging from 1 ~ 10 *kPa* and thicknesses *h* from 5∼10 *nm*. (PDF)

S3 Table Values of *β* for Young’s modulus *E* ranging from 1 ~ 10 *kPa* and thicknesses *h* from 5 ~ 10 *nm*. (PDF)
