# Supplementary material for: Modeling of the axon membrane skeleton structure and implications for its mechanical properties
Source: PLoS Comput Biol. 2017 Feb 27;13(2):e1005407. doi: 10.1371/journal.pcbi.1005407 (PMC5348042; doi:10.1371/journal.pcbi.1005407)
Supplement: S1 Text — (DOCX) [file pcbi.1005407.s001.docx]

**S1 Text**

**Indentation of a neo-Hookean half space and a thin-walled cylinder using a conical indenter with a spherical tip**

Large deformations of cell surface membranes can be described reasonably well using the neo-Hookean constitutive model based on a convex strain-energy function $\text{ψ}$ [13,22,23]. We employ the isochoric deformation gradient $\bar{\mathbf{F}}=J^{-1/3}\mathbf{F}$, and similarly the isochoric right Cauchy-Green tensor $\bar{\mathbf{C}}=J^{-2/3}\mathbf{C}$, where $\mathbf{C=F}^{\text{T}}\mathbf{F}$and $J$ is the Jacobian, the determinant of the deformation gradient $\mathbf{F}$. For the neo-Hookean model

$\text{ψ = }\frac{1}{c}\left(J-1\right)^{2}+\frac{\mu}{2}\left(\bar{I}_{1}-3\right),$ (S1)

where $c=6\left(1-2\nu\right)/E=2/\kappa$, $E$is the initial Young’s modulus, $\nu$ is the Poisson’s ratio, and $\kappa$ is the initial bulk modulus of the material. In the case of incompressibility, $c$ degenerates to a nonphysical, positive penalty parameter used to enforce incompressibility. The parameter $\mu$ is the initial shear modulus, and$\bar{I}_{1}=\text{tr}\bar{\mathbf{C}}$ is the first invariant of the isochoric right Cauchy-Green tensor. From the deformation gradient, we calculate Cauchy stress $\mathbf{\sigma}$ as

$\mathbf{\sigma}=\frac{2}{J}\mathbf{F}\frac{\partial\text{ψ}}{\partial\mathbf{C}}\mathbf{F}^{\text{T}}$, (S2)

Applying the nearly incompressible neo-Hookean material model, we simulate indentation with a conical indenter (with a blunt tip of a specific spherical curvature) of both (*i*) a half-space, and (*ii*) a thin-walled cylinder using finite element analyses in ANSYS workbench 14.0 (Canonsburg, PA).

**Indentation of a neo-Hookean rectangular cuboid**

We employ finite element analysis (FEA) to obtain the force-indentation $\left(F-d\right)$ relationship $F=g\left(E,\nu\right)d^{\alpha\left(E,\nu\right)}$ that is valid for the indentation of the pyramidal neuron soma with an atomic force microscope (AFM) indenter. By fitting the $\left(F-d\right)$ curve to AFM experimental data, we then estimate the Young’s modulus $E$ of the soma plasma membrane assuming that $\nu\approx0.5$. The diameter of the soma is approximately $20 \mu m$ and its thickness is approximately $10 \mu m$[29]. The indenter is a square right pyramid with a height between $2.5$ and $8.0 \mu m$, a semi-included angle of approximately $20^{\circ}$, and a nominal tip radius of $20 nm$, as provided by the manufacturer (see Figure A in S4 Fig). The maximum indentation was $\sim200 nm$ but only $\sim100 nm$ of the indentation was used to determine the Young’s modulus of the soma.

To validate the FEA, we first consider indentation of an infinite elastic half-space and compare the analytical and numerical solutions. For rigid indenters with blunt tips (Figure A and B in S4 Fig) indenting a nearly incompressible elastic half-space $\left(E=2 kPa, \nu\approx0.5\right)$ without adhesion the force-indentation relationship is (Lin et al., 2006; Costa and Yin, 1999; Rico et al., 2005)

$F=\frac{2E}{1-\nu^{2}}\left\{ad-m\frac{a^{2}}{\tan\theta}\left[\frac{\pi}{2}-\arcsin\left(\frac{b}{a}\right)\right]-\frac{a^{3}}{3R}+\left(a^{2}-b^{2}\right)^{1/2}\left[m\frac{b}{\tan\theta}+\frac{a^{2}-b^{2}}{3R}\right]\right\}$, (S3a)

where $\theta$ is the semi-included tip angle, $R$ is the tip radius, $b$ is the radius at which the conical or pyramidal indenter transitions to the spherical tip, $a$ is the contact distance, and $m$ is a constant that characterizes the cone $\left(m=1/2\right)$ and the pyramid $\left(m=\sqrt{2}/\pi\right)$ (Lin et al., 2006). The contact length $a$ depends on the indentation depth as

$d+\frac{a}{R}\left[\left(a^{2}-b^{2}\right)^{1/2}-a\right]-n\frac{a}{\tan\theta}\left[\frac{\pi}{2}-\arcsin\left(\frac{b}{a}\right)\right]=0$, (S3b)

where $n$ is a constant that characterizes the cone $\left(n=1\right)$ and the pyramid $\left(n=2^{3/2}/\pi\right)$ (Lin et al., 2006). The force-indentation expression given above is valid for $d>b^{2}/R$. For lower values of $d$, where only the spherical tip of the indenter contacts the half-space, the Hertz contact model

$F=\frac{4ER^{1/2}}{3\left(1-\nu^{2}\right)}d^{3/2}$, (S4)

applies as this models a spherical rigid body indenting an elastic half-space (Johnson, 1985). We plot in Figure C in S4 Fig the analytically derived $F-d$ curves for a spherical indenter with a radius of $20 nm$, and for a conical and a pyramidal indenter with a semi-included angle of $\theta=20^{\circ}$ and a blunt tip of radius $20 nm$. We observe that the conical and pyramidal indenters with a blunt tip of the same radius produce similar force-indentation relationships.

To derive a numerical solution, we model the elastic half-space as a rectangular cuboid with dimensions $10 \mu m \times10 \mu m \times5 \mu m$, meshed with SOLID 185 hexahedral elements, and fixed in all degrees of freedom at the bottom surface opposite the indenter. We determined the final mesh density using a convergence analysis. The rigid conical indenter has a height of $250 nm$, a semi-included angle of $\theta=20^{\circ}$, and a tip radius of $20 nm$. We apply frictionless contact between the rigid indenter and the elastic half-space using elements TARGE 170 and CONTA 174, respectively. We generate a force-displacement curve using the total reaction force during the controlled indentation. As we see the numerical and analytical solutions are almost identical (Figure C in S4 Fig) along the entire indentation depth. Also, as expected, the analytical and the numerical results match the analytical result for a spherical indenter up to $d=R$ (Figure C in S4 Fig).

After validating our FE analysis for the nearly incompressible elastic half-space, we use the same numerical approach (a nearly incompressible neo-Hookean half-space, SOLID 185 hexahedral elements, frictionless contact using TARGE 170 and CONTA 174) and we compare the analytical $F-d$ expression for the elastic half-space with the numerical solution for the corresponding neo-Hookean half-space for the same conical indenter. We use a volume $10 \mu m \times10 \mu m \times5 \mu m$ with $\nu\approx0.5$ and elastic modulus of $2 kPa$ (i.e. $E=2 kPa, \nu\approx0.5$ is equivalent to$\mu=2/3kPa$, $c<<\mu$). Since the force-indentation response for a conical and a pyramidal indenter are similar, we use a conical indenter to avoid singularities caused from stress concentrations at the contact between the indented space and the edges of the pyramid. The displacement field along the z-direction is estimated using FEA (S5 Fig). The response of the linear elastic and the neo-Hookean estimates are similar at small indentations but the neo-Hookean model is softer at larger indentations (S6 Fig).

We also investigate the effect of a rigid substrate on the force-indentation curve during indentation of a neo-Hookean volume ($1 \mu m \times1 \mu m \times h$, where $h$ is the thickness), by a conical indenter with a blunt tip. We reduce the thickness of the neo-Hookean material from $500 nm$ to $100 nm$, while keeping the maximum indentation constant at $50 nm$, and compare the resulting force-indentation curves with the theoretical linear elastic solution cf. S7 Fig. We observe that at $h=500 nm$ the $F-d$ curve generated using FEA is similar to the one shown in S6 Fig and it remains below the elastic response above $15 nm$ as we expect for a neo-Hookean material. However, when we reduce the thickness to $300 nm$ the response is stiffer than the linear elastic response, clearly indicating the effect of the substrate at indentations larger than $\sim30 nm$. We observe the same effect for the simulation including a $200 nm$ thickness for indentations larger than $\sim20 nm$. Note that both limits are approximately $10\%$ of the corresponding material thickness. We thus assume that for the neo-Hookean volume of $10 \mu m \times10 \mu m \times5 \mu m$ and for indentations up to $200 nm$ the substrate does not affect the results.

Finally, we obtain the force-indentation relationship for a nearly incompressible neo-Hookean half-space $\left(\nu\approx0.5\right)$, indented using a rigid cone with a blunt tip (radius of $R=20 nm$ and semi-included angle $\theta=20^{\circ}$) with the following approach. First, we assumed that the $F-d$ expression is of the form $F=A(E)d^{\alpha\left(E\right)}$, cf. (Lin et al., 2006). We then ran finite element simulations for the specific indenter and for initial Young’s moduli ranging from $0.5$ to $10 kPa$. For each simulation we track the indentation depth and extract the corresponding force for indentations up to $200 nm$, and then use a least-square fitting method to obtain the parameters $A\left(E\right)$ and $\alpha\left(E\right)$. An example for $E=2 kPa$ is shown in S8 Fig. The results for different initial Young’s moduli are shown in S9 Fig. We found that $A\left(E\right)=7.95\times10^{-3} E$ (Figure A in S9 Fig) while the power term is independent of the initial Young’s modulus $\alpha\left(E\right)=1.46$. Hence, the relation between the load $F$applied with this specific conical indenter and for large indentations $d$ into a nearly incompressible neo-Hookean half space is described by the expression

$F=\left(7.95\times10^{-3}E\right) d^{1.46}$, (S5)

We note that the relationship (S5) is valid when the indentation $d$ is measures in $nm$, the initial Young’s modulus $E$ in $kPa$, and the force $F$ in $pN$. We apply expression (S5) to estimate the Young’s modulus $E$ of the soma using our AFM indentation measurements. We also note the similarity between our solution and the analytical solution for elastic half-space indentation which predicts that the $F-d$ power law for spherical indenters is $3/2$.

**Indentation of a thin-walled cylinder**

To estimate the Young’s modulus $E$ of the plasma membrane in dendrites and unmyelinated axons, we follow a similar approach as above. We model the surface membrane of unmyelinated axons and dendrites as a thin-walled, nearly incompressible$\left(\nu\approx0.5\right)$, neo-Hookean cylinder with a length $L=2 \mu m$, an external radius $R_{c}=0.5 \mu m$, and a wall thickness $h$ (that will be determined below). We clamp the two axial ends of the cylinder in all degrees of freedom. We mesh the volume with SOLID 185 hexahedral elements (final mesh density again determined using a convergence analysis) and modeled the conical indenter as a rigid body with a tip radius $R=20 nm$ and a semi-included angle $\theta=20^{\circ}$. Again, we apply frictionless contact between the rigid indenter and the cylinder using TARGE 170 and CONTA 174, respectively. We aim to estimate the force-indentation $\left(F-d\right)$ relationship of the form

$F=B\left(E\right) d^{\beta\left(E\right)}$, (S6)

for a specific wall thickness $h$, similar to equation (S5), and then use AFM measurements to extrapolate $E$. We generate force-displacement curves by applying indentations and calculating the total reaction force from the model.

First, we select a cylinder with a wall-thickness $h=10 nm$ and an initial elastic modulus of $2 kPa$, and simulate indentation up to $200 nm$, cf. S10 Fig. Our results reveal that for indentations less than $30 nm$, there is a relatively rapid increase in required force due to the induced curvature both along and perpendicular to the longitudinal principal axis. The result agrees with results shown in (Vaziri and Mahadevan, 2008). Neglecting the indentation depth smaller than $30 nm$ and assuming that in large deformation the load and indentation follow the relationship of $F=B d^{\beta}$, we obtain that $\beta=1.37$ and $B=8.21\times10^{-3} pN/nm^{1.37}$ by employing curve fit in S11 Fig.

In addition to its material properties, the wall-thickness of a cylindrical shell is expected to influence the $F-d$ parameters $B\left(E,h\right)$ and $\beta\left(E,h\right)$. We curve fit a series of numerical $F-d$ results (as explained above) to determine how $B\left(E,h\right)$ and $\beta\left(E,h\right)$ depend on the wall-thickness and the initial Young’s modulus (within relevant limits). We provide examples of the resulting force-indentation curves (i) for initial $E=2 kPa$ and for different wall-thicknesses in Figure A in S12 Fig, and (ii) for fixed wall-thickness $h=10 nm$ and initial elastic modulus ranging from $1 kPa$to $10 kPa$ in Figure B in S12 Fig. We note that for wall-thicknesses less than $10 nm$, and for initial Young’s moduli below $10 kPa$, the initial abrupt increase in required indenting force is limited to less than $30 nm$ indentation. In AFM experiments, this length ($30 nm$) corresponds to the tip making contact with the surface membrane, which is not well-defined. Because of this, we curve-fit the numerical results beyond the $30 nm$ initial displacement. From this curve-fitting we obtain parameters $B$ and $\beta$ for the expression $F=B d^{\beta}$ for each specific case. We provide the results in S1 and S2 Table, respectively. From the results shown in S2 Table, we conclude that $\beta$ varies approximately from 1.34 to 1.37 when the thickness ranges from $5 nm$ to $10 nm$ and the initial Young’s modulus varies from $1$ to $10 kPa$. This means that, within these thickness limits, $\beta$ is almost constant and close to the value for a rigid sphere indenting an elastic half-space.

Finally, we specifically determine the relation between *E* and $B\left(E\right)$, cf. Figure A in S13 Fig and between $E$and $\beta\left(E\right)$, cf. Figure B in S13 Fig for the case $h=10 nm$, since we consider that the thickness of the membrane skeleton is ~$10 nm$. We find that $B(E)=4.41\times10^{-3}E$ and $\beta=1.37$. Hence, we conclude that the force-indentation relationship for a conical indenter, with a semi-included angle $\theta=20^{\circ}$ and a blunt tip of radius $R=20 nm$, indenting a nearly incompressible neo-Hookean thin-walled cylinder of wall-thickness $h=10 nm$ at large indentations is

$F=\left(4.41\times10^{-3}E\right)d^{1.37}$. (S7)

We note that the relationship (S7) is valid when the indentation $d$ is measured in $nm$, the initial Young’s modulus $E$ in $kPa$, and the force $F$ in $pN$.

**Bending rigidity of actin filaments**

To compute the bending rigidity of actin filaments we follow the strategy outlined in [37]. The bending rigidity $\kappa_{bend}$ of semi-flexible filaments can be derived from thermal oscillations [20] via the expression $\kappa_{bend}=\frac{K_{B}TL^{3}}{3\left\langle\delta u\left(L\right)^{2}\right\rangle}$, where $L$ is the total length of the actin filament, $T$ is the temperature in Kelvin $\left(K\right)$, $K_{B}=1.3807\times10^{-23} JK^{-1}$ is the Boltzmann constant, $\delta u\left(L\right)=u\left(L\right)-\left\langle u\left(L\right)\right\rangle$is the normal deviation of the filament end measured from its average position (Jones et al., 2005). We recorded the end displacement of an actin filament of length $L\simeq1.36 \mu m$ and then we plotted the probability distribution of the recorded values. We calculated the mean value $\left\langle\delta u\left(L\right)^{2}\right\rangle^{1/2}\simeq0.22 \mu m$ by fitting the results with a Gaussian distribution (S15 Fig). The corresponding bending rigidity is $7.1\times10^{-26} Nm^{2}$ which is close to the reported experimental value of $7.3\times10^{-26} Nm^{2}$ [38, 39].

**References**

Lin DC, Dimitriadis EK, Horkay F. Robust Strategies for Automated AFM Force Curve Analysis—I. Non-adhesive Indentation of Soft, Inhomogeneous Materials. Journal of Biomechanical Engineering. 2006;129(3):430-40.

Costa KD, Yin FCP. Analysis of Indentation: Implications for Measuring Mechanical Properties With Atomic Force Microscopy. Journal of Biomechanical Engineering. 1999;121(5):462-71.

Rico F, Roca-Cusachs P, Gavara N, Farré R, Rotger M, Navajas D. Probing mechanical properties of living cells by atomic force microscopy with blunted pyramidal cantilever tips. Physical Review E. 2005;72(2):021914.

Johnson KL. Contact mechanics. Cambridge Cambridgeshire ; New York: Cambridge University Press; 1985. xi, 452 p. p.

Vaziri A, Mahadevan L. Localized and extended deformations of elastic shells. Proc Natl Acad Sci U S A. 2008;105(23):7913-8.

Jones CW, Wang JC, Briehl RW, Turner MS. Measuring Forces between Protein Fibers by Microscopy. Biophysical Journal. 2005;88(4):2433-41.
