# Supplementary figures and images for: Modeling of the axon membrane skeleton structure and implications for its mechanical properties
Source: PLoS Comput Biol. 2017 Feb 27;13(2):e1005407. doi: 10.1371/journal.pcbi.1005407 (PMC5348042; doi:10.1371/journal.pcbi.1005407)

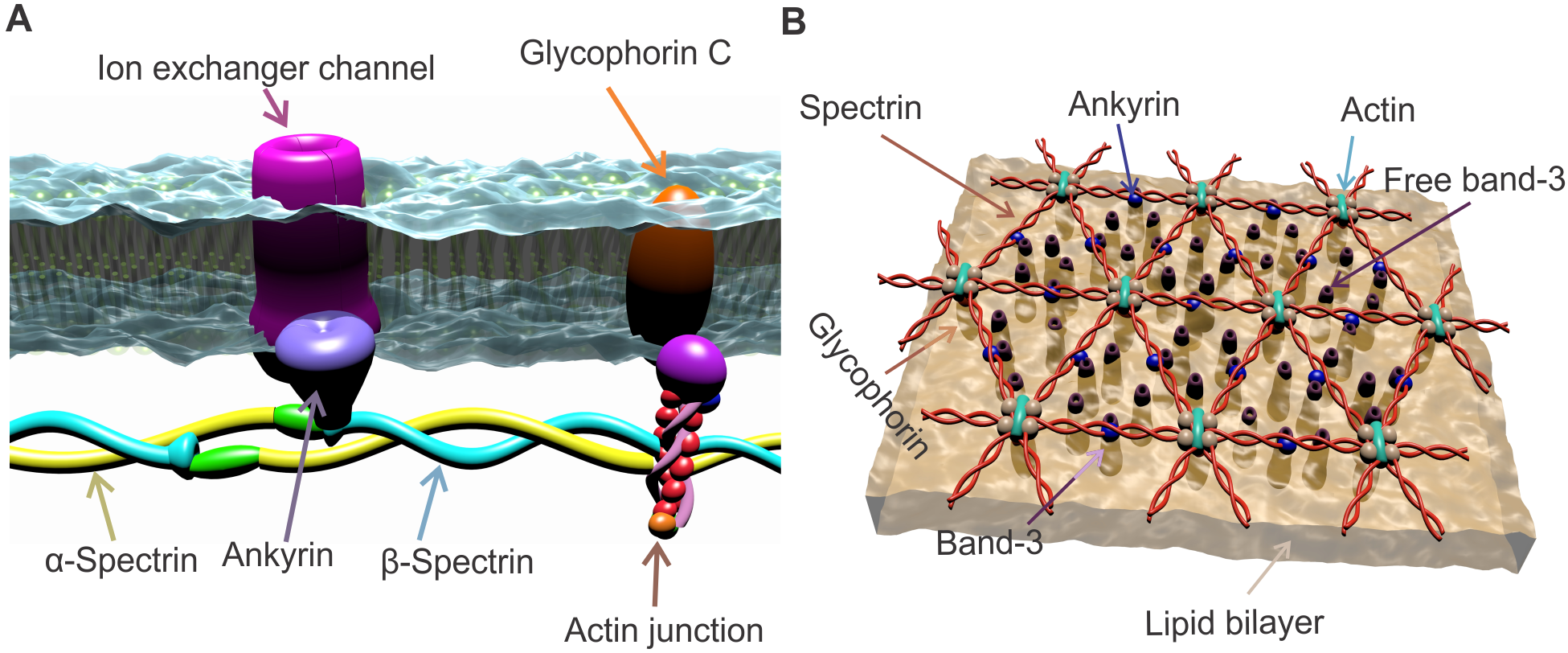

Supplement: S1 Fig — (A) Anchoring of the lipid bilayer to the membrane skeleton. Ankyrin binds to the 15th repeat of β-spectrin near its carboxyl terminus and to an anion exchanger band-3 in RBCs. α-spectrin and β-spectrin filaments are connected at actin junctions. In the axon plasma membrane, ankyrin binds to voltage-gated sodium channels (Nav). In RBCs, the NH2-terminal of β-spectrin binds to protein 4.1 which forms a membrane anchoring complex with glycophorin C [7]. (B) Illustration of the RBC membrane skeleton comprising stretched spectrin tetramers connected at actin junctions and exhibiting a six-fold two-dimensional symmetry. The lipid bilayer is anchored to the membrane skeleton at actin junctions by glycophorin C and near the middle of each spectrin tetramer by ankyrin which is then connected to an anion exchanger band-3 protein [7, 59]. (TIF) [file pcbi.1005407.s002.tif]

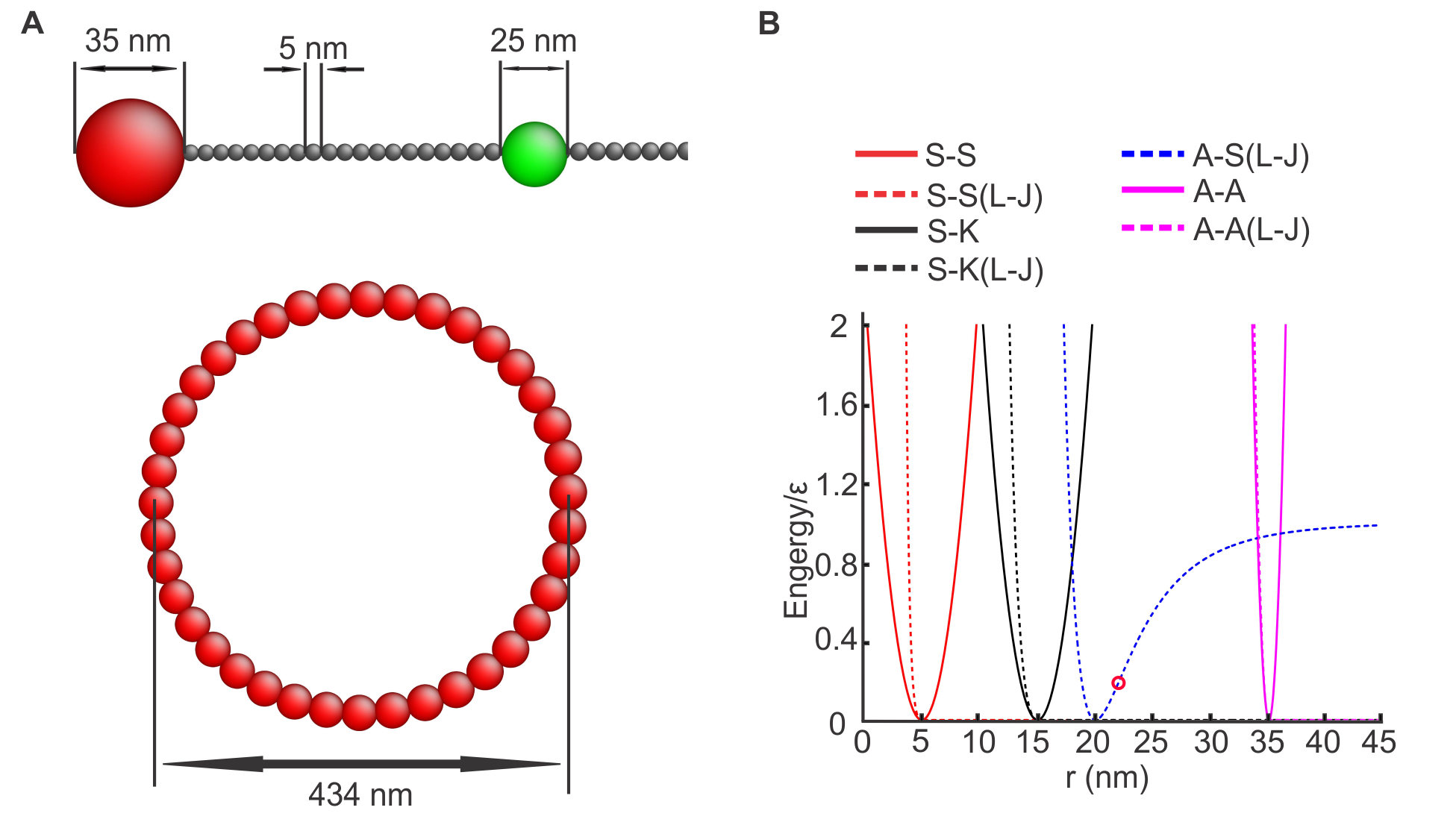

Supplement: S2 Fig — (A) Illustration of particles and connections involved in the model. Red particles represent actin junctions, gray particles represent spectrin subunits, and green particles represent ankyrin junctions. The diameter of the axon model is 434 nm. (B) The solid lines represent harmonic potentials applied between neighboring spectrin particles (S-S) of the same spectrin filament, spectrin and ankyrin (S-K), and between neighboring actin particles (A-A) in the actin rings. Dashed lines depict repulsive Lennard-Jones (L-J) potentials which represent steric repulsions between all particles used in the simulation. The blue dashed depicts the L-J potential applied between actin and spectrin (A-S). Note that the linkage between A-S is breakable at the inflexion distance of the potential marked with a red circle. (TIF) [file pcbi.1005407.s003.tif]

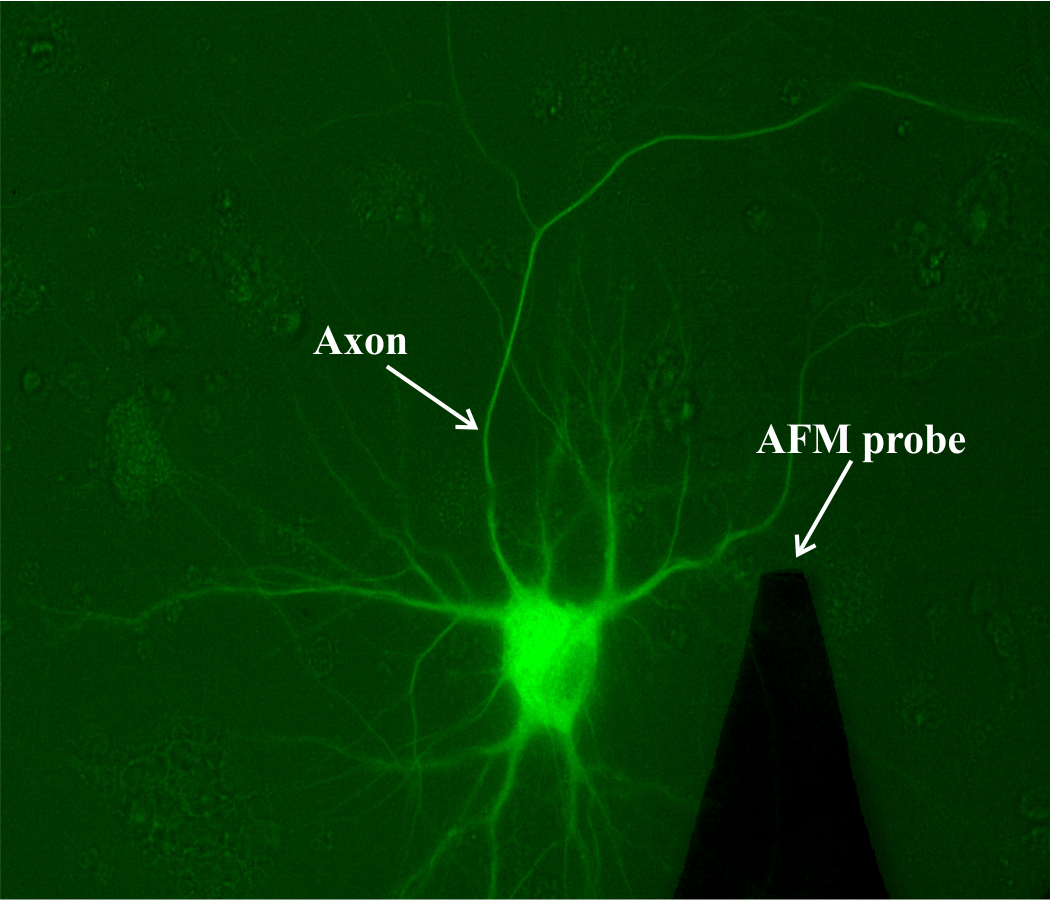

Supplement: S3 Fig — (TIF) [file pcbi.1005407.s004.tif]

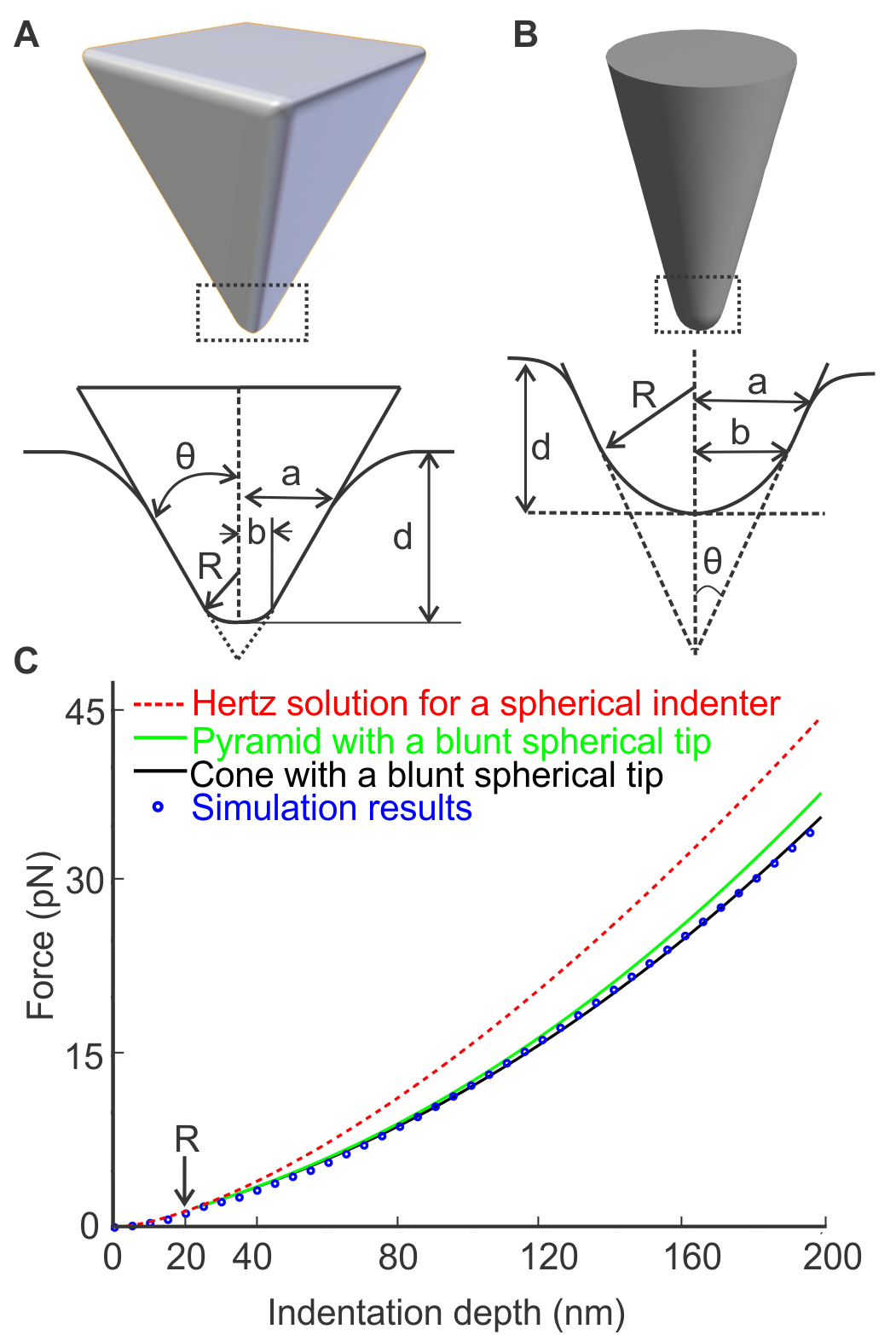

Supplement: S4 Fig — (A) A pyramidal indenter with a blunt tip of radius 20 nm and a semi-included angle of 20°. (B) A conical indenter with a blunt tip of radius 20 nm and a semi-included angle of 20°. (C) Comparison between the analytically derived F − d curve (black solid line) for an elastic half-space indented by the conical indenter described in B and the corresponding FE (blue circles) results of indentation of an elastic 10 μm×10 μm×5 μm cuboid with E = 2 kPa and ν ≈ 0.5. The analytically derived F − d curves for the pyramidal indenter described in A and for a spherical indenter of radius 20nm are also shown as a solid green line and a red dashed line respectively. The F − d indentation curves for a conical and a pyramidal indenter with a blunt tip, which have the same semi-included angles and the same radii of the tips, are similar when indenting a half-space. (TIF) [file pcbi.1005407.s005.tif]

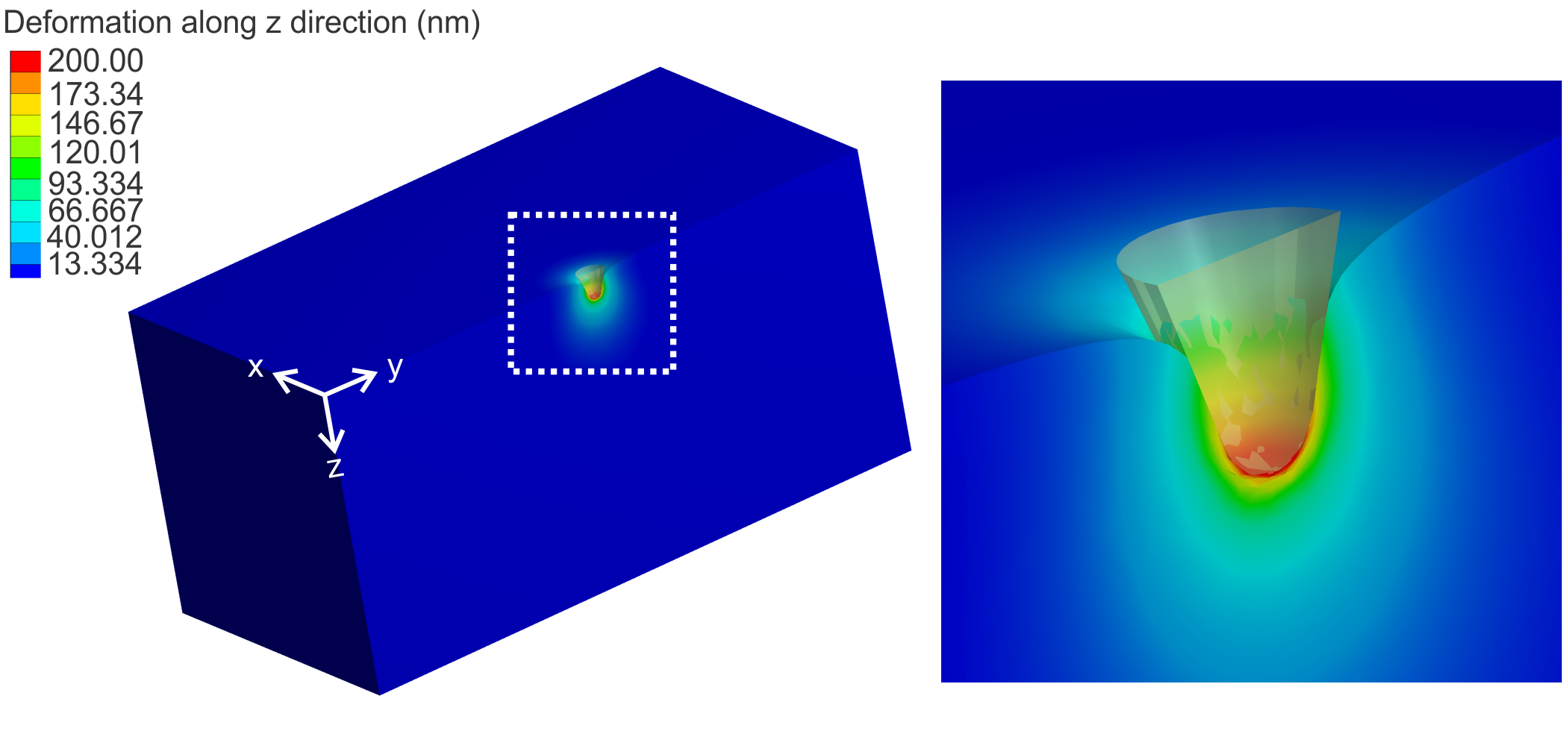

Supplement: S5 Fig — The color map represents the vertical displacement (z-axis) field in nm. (TIF) [file pcbi.1005407.s006.tif]

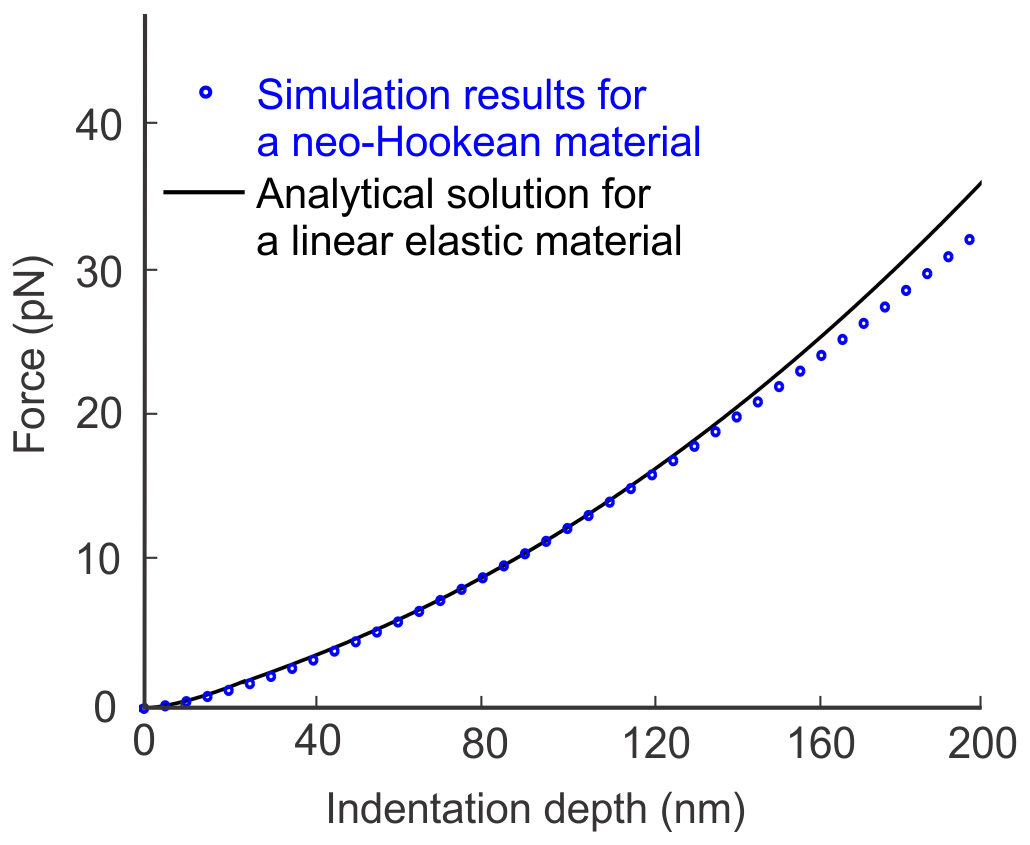

Supplement: S6 Fig — Comparison of indentation simulation results for a neo-Hookean 10 μm×10 μm×5 μm cuboid of E = 2 kPa and ν = 0.5 with the classic Hertz solution for a rigid conical indenter with a blunt tip of 20 nm radius and a semi-included angle of 20° for indentations up to 200 nm. (TIF) [file pcbi.1005407.s007.tif]

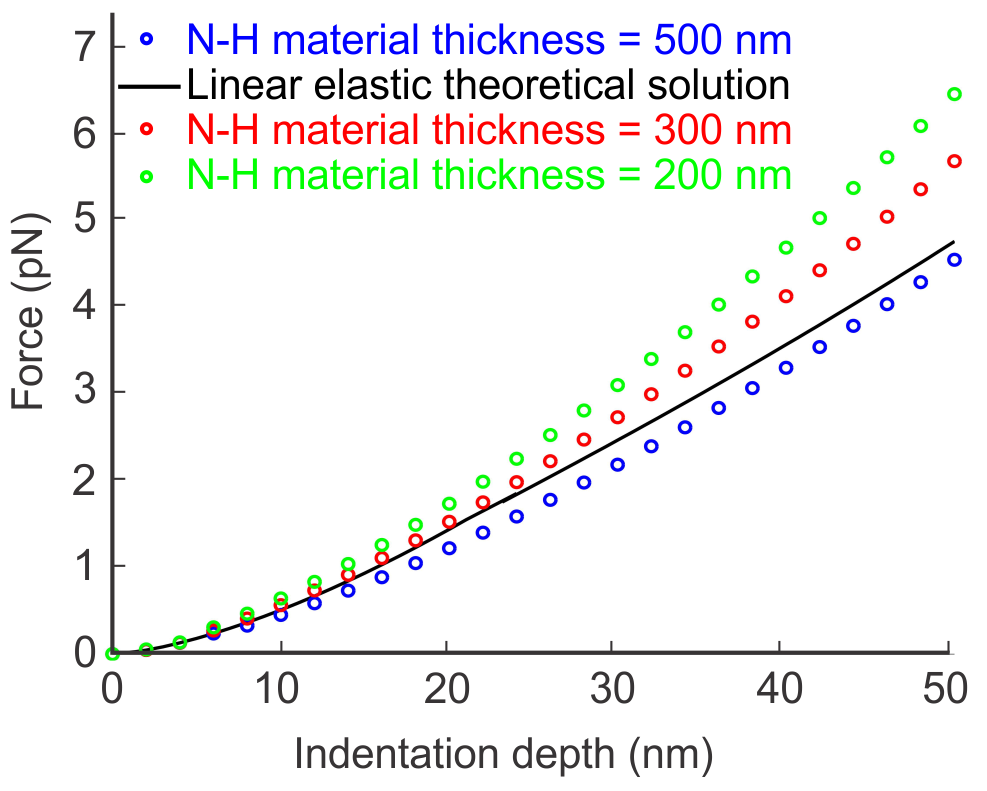

Supplement: S7 Fig — (TIF) [file pcbi.1005407.s008.tif]

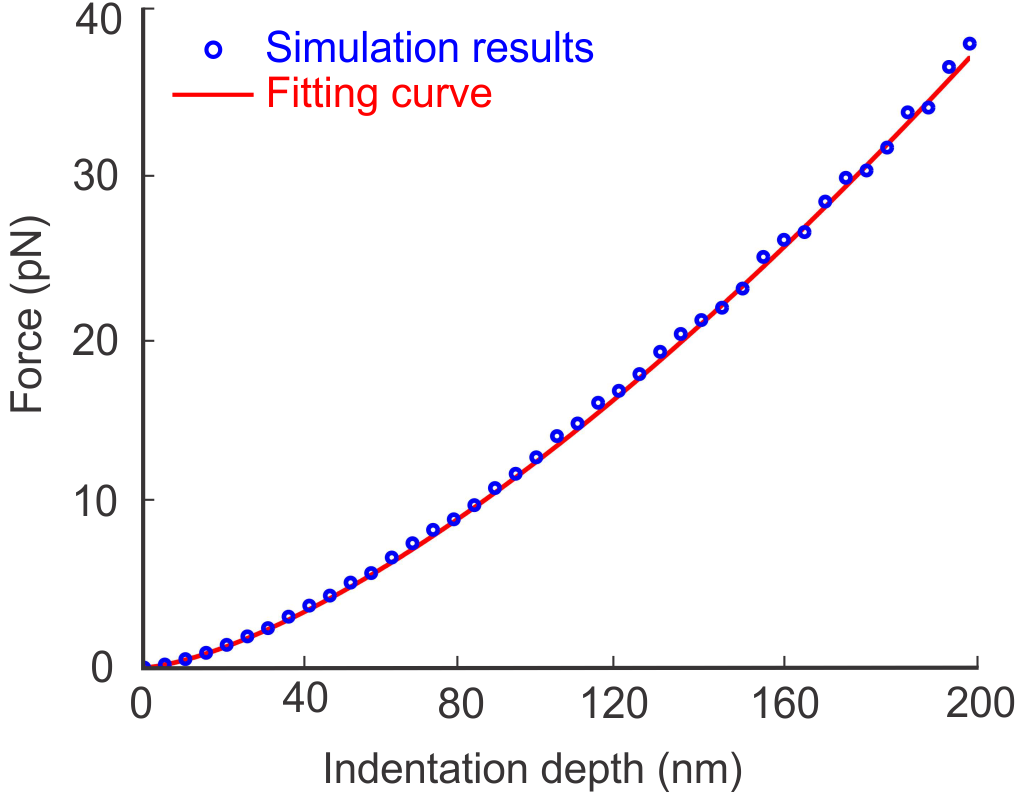

Supplement: S8 Fig — We assume the fitting curve is of the form F = A dα. (TIF) [file pcbi.1005407.s009.tif]

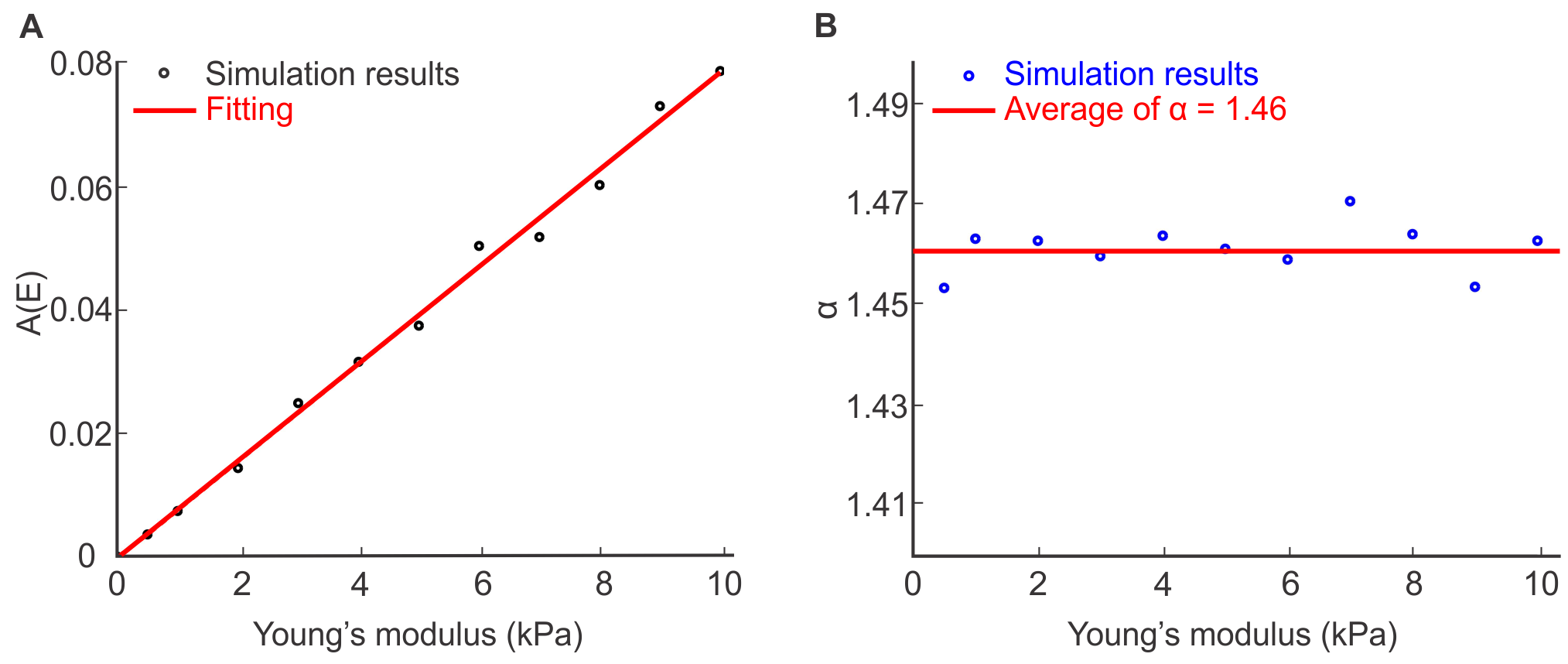

Supplement: S9 Fig — Relationships between (A) E and A(E) and (B) between E and α within a range of Elastic moduli from 0.5 kPa to 10 kPa. (TIF) [file pcbi.1005407.s010.tif]

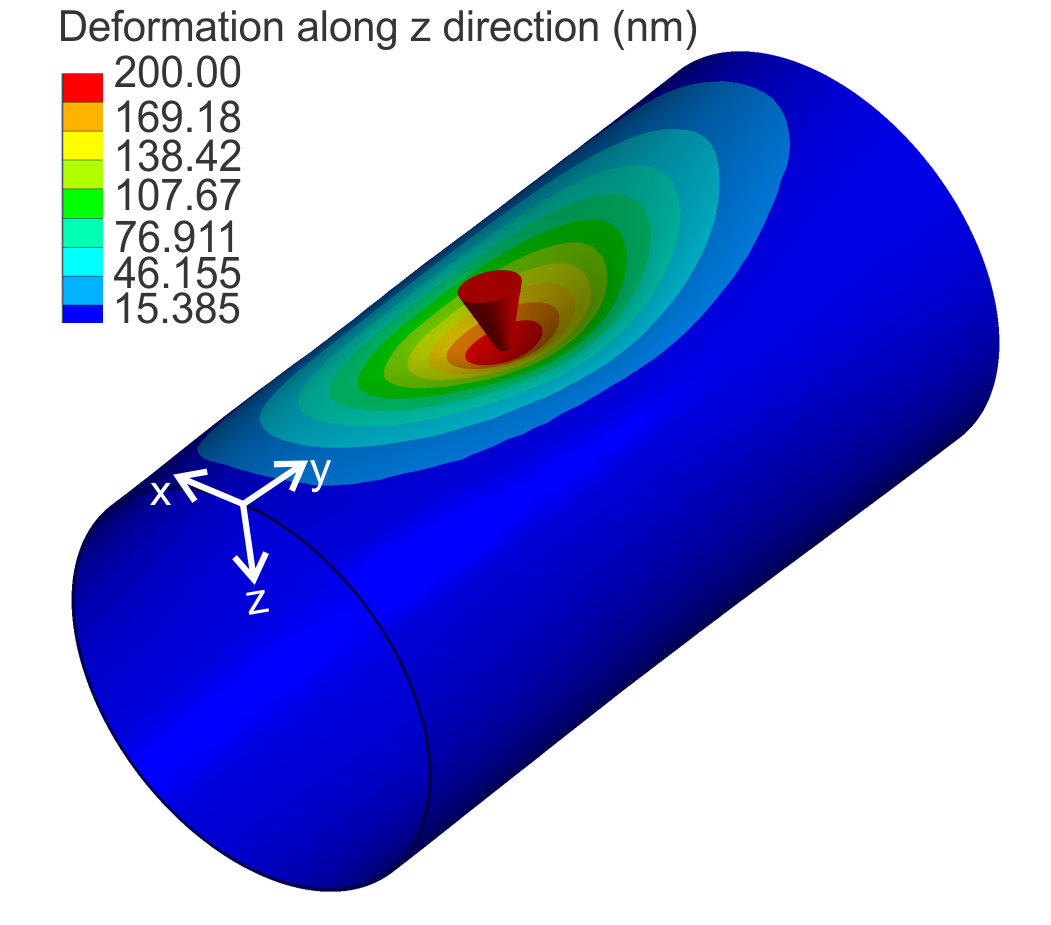

Supplement: S10 Fig — The color map represents the vertical (z-direction) displacement field measured in nm. (TIF) [file pcbi.1005407.s011.tif]

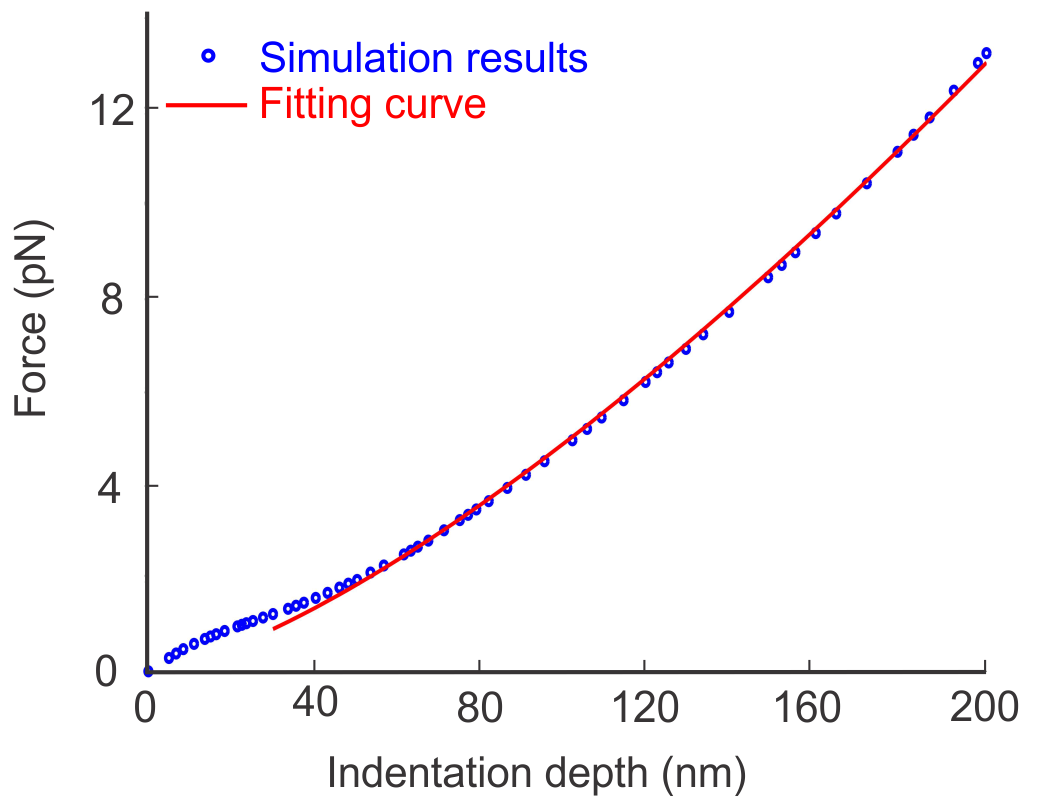

Supplement: S11 Fig — (TIF) [file pcbi.1005407.s012.tif]

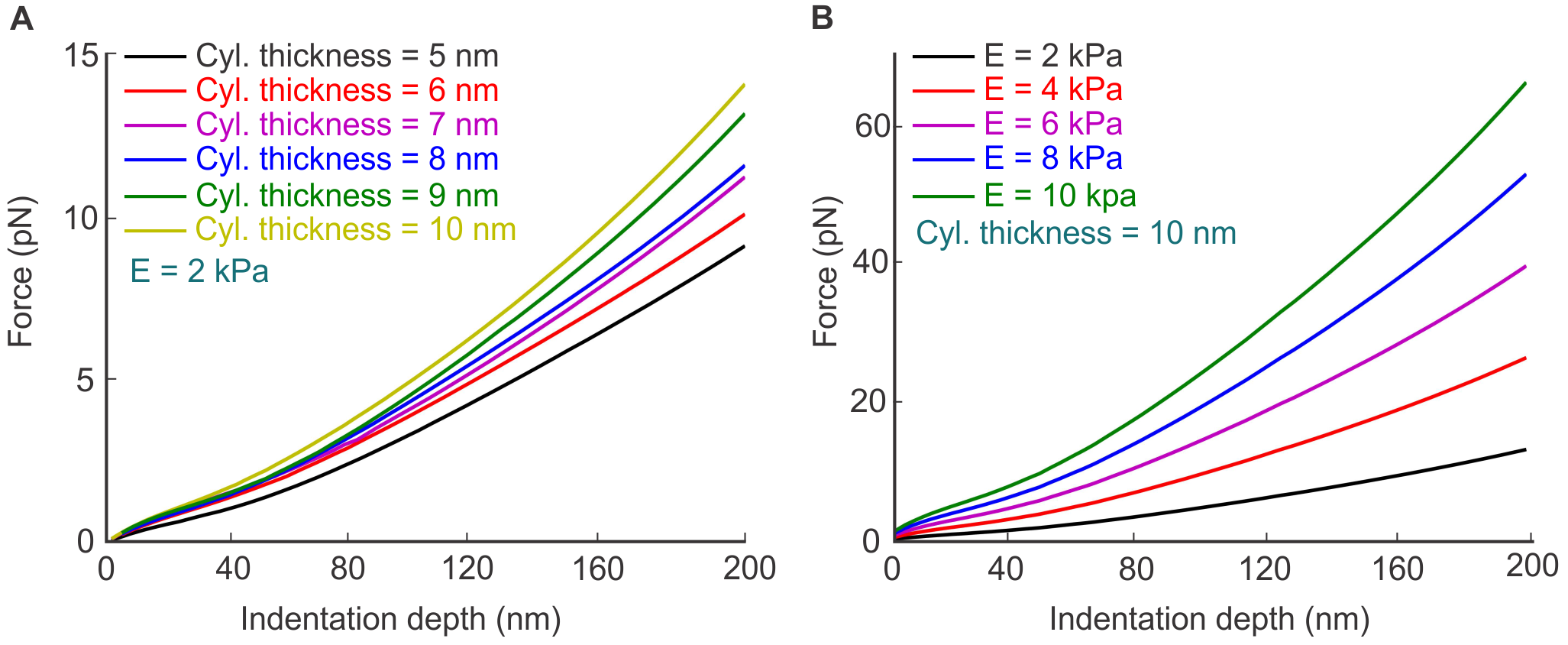

Supplement: S12 Fig — Comparison of simulation results at indentations up to 200 nm, (A) a range of thickness from 5 nm to 10 nm with fixed E = 2 kPa, (B) a range of E from 2 kPa to 10 kPa with fixed thickness h = 10 nm. (TIF) [file pcbi.1005407.s013.tif]

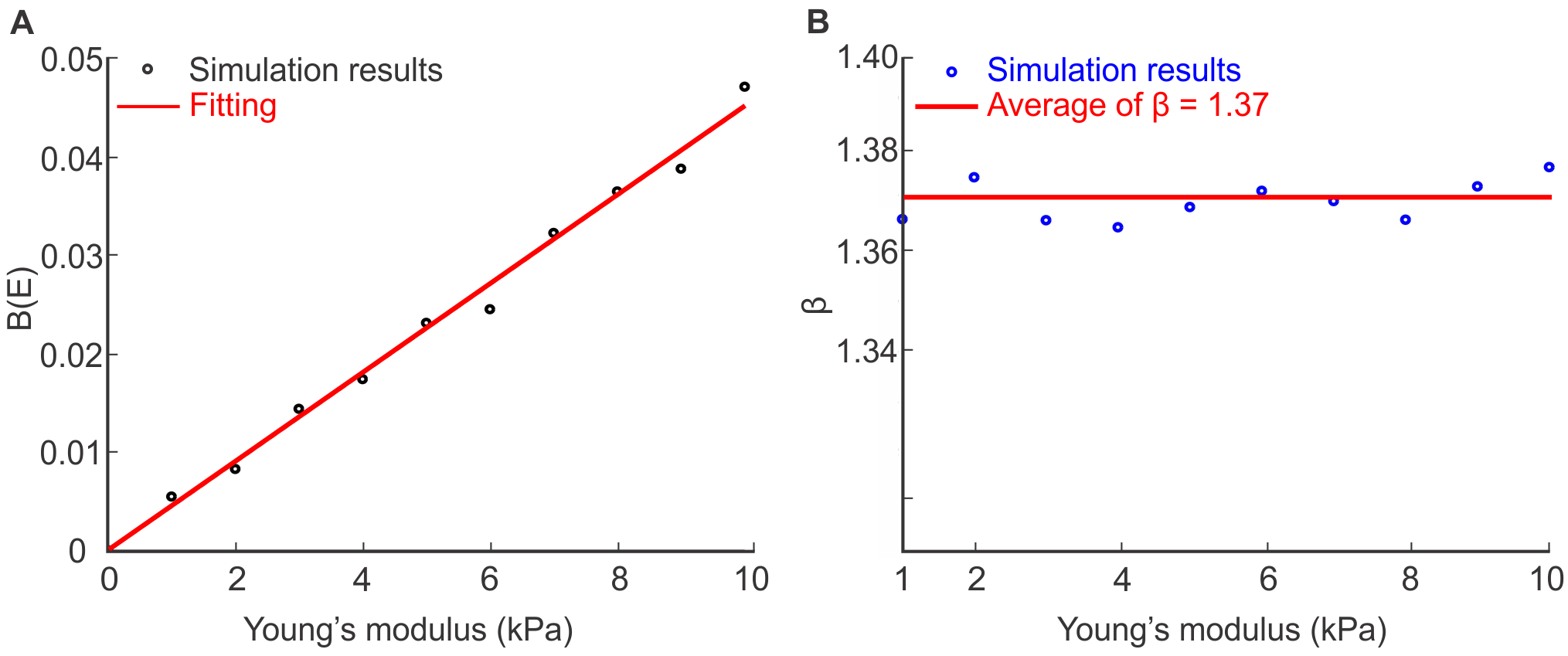

Supplement: S13 Fig — Relationships (A) between E and B(E), and (B) between E and β determined from a range of assumed elastic moduli from 1 to 10 kPa for cylinder wall-thickness h = 10 nm. (TIF) [file pcbi.1005407.s014.tif]

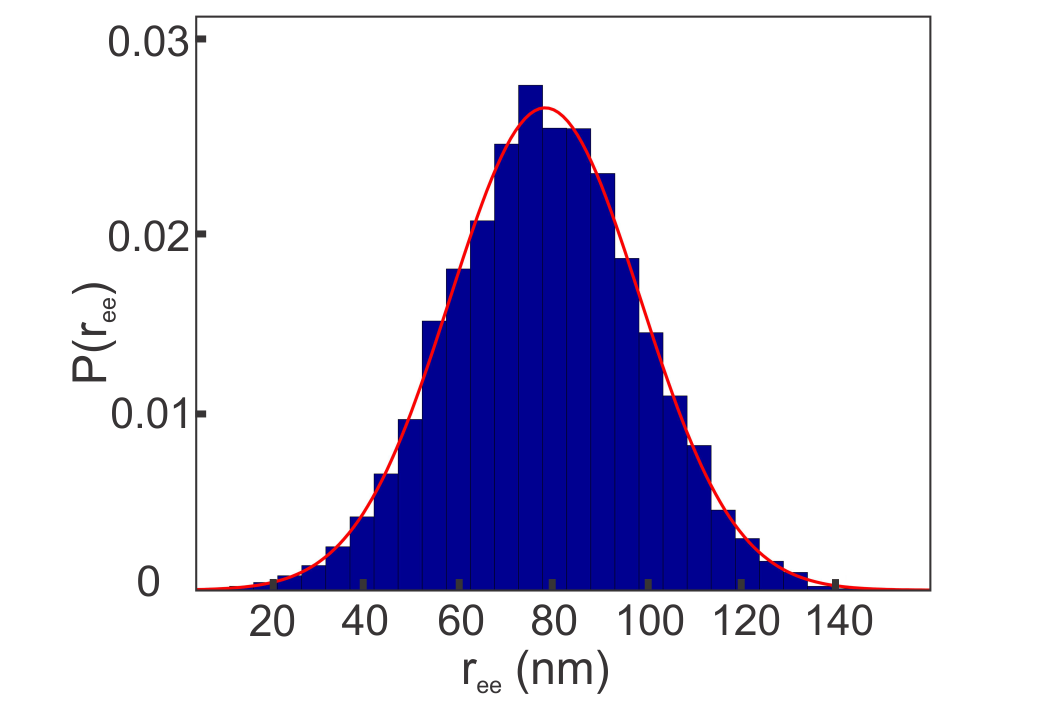

Supplement: S14 Fig — The associated normalized Gaussian probability density (red line) is also shown. (TIF) [file pcbi.1005407.s015.tif]

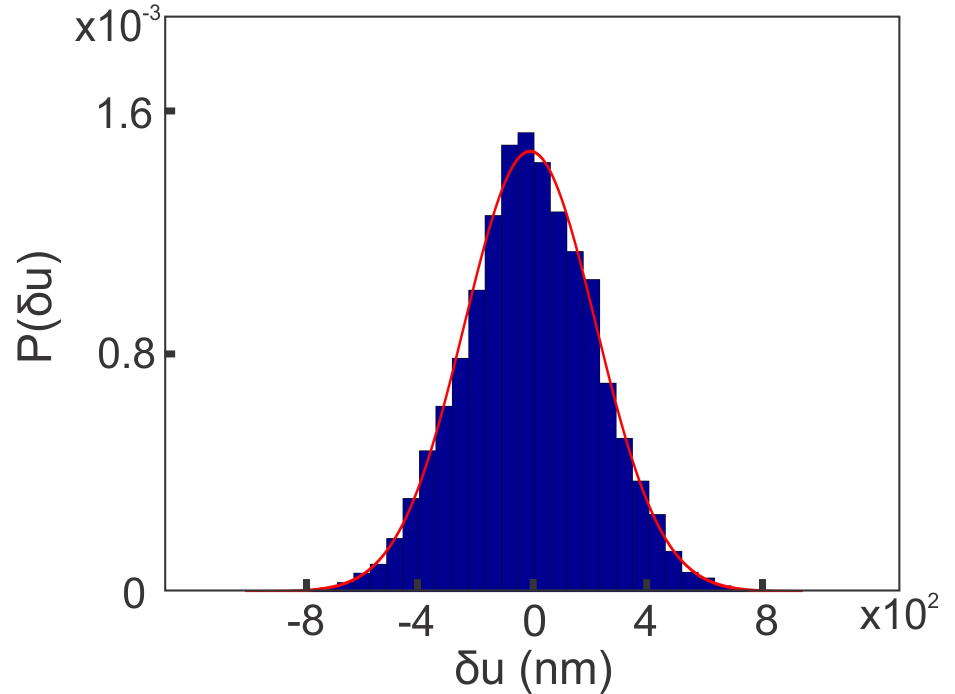

Supplement: S15 Fig — The associated normalized Gaussian probability density is shown in red. (TIF) [file pcbi.1005407.s016.tif]

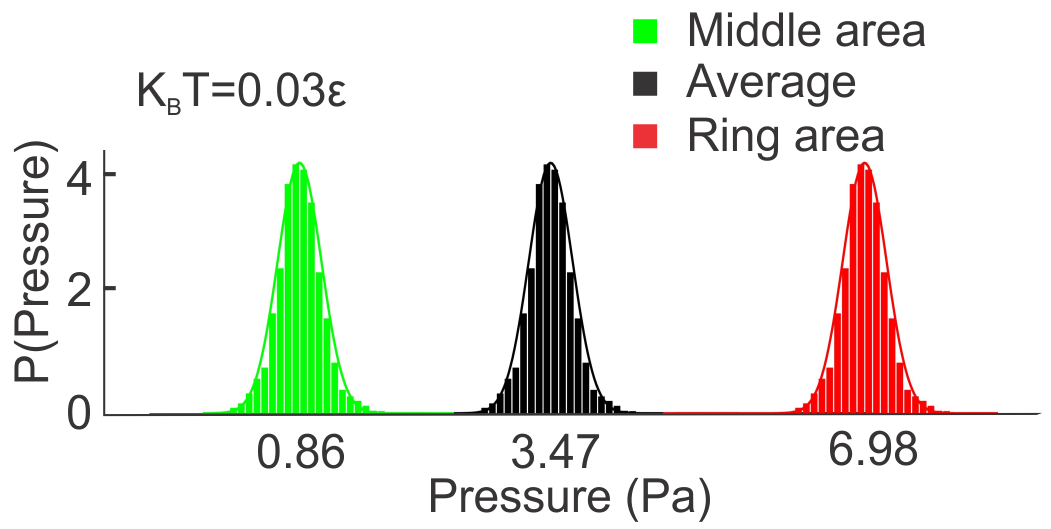

Supplement: S16 Fig — The associated normalized Gaussian probability densities are also shown. (TIF) [file pcbi.1005407.s017.tif]
