# Supplementary material for: Modeling of the axon membrane skeleton structure and implications for its mechanical properties
Source: PLoS Comput Biol. 2017 Feb 27;13(2):e1005407. doi: 10.1371/journal.pcbi.1005407 (PMC5348042; doi:10.1371/journal.pcbi.1005407)
Supplement: S1 Table — (PDF) [file pcbi.1005407.s018.pdf]

S1 Table. Potentials and corresponding parameters used in modeling of the axon membrane skeleton.

<table>
<tr><th colspan="4">Potentials</th></tr>
<tr><td colspan="2">Spring potential</td><td colspan="2">$U_{Spring}(r) = 1/2\, K(r - r_0)^2$</td></tr>
<tr><td colspan="2">Lennard-Jones potential</td><td colspan="2">$U_{LJ}(r) = 4\mathrm{E}\left[(S/r)^{12} - (S/r)^{6}\right]$</td></tr>
<tr><td colspan="2">Bending FENE potential</td><td colspan="2">$U_{bending} = -\frac{1}{2} k_b\, \Delta\theta_{max}\, ln\left[1 - \left(\frac{\theta - \theta_0}{\Delta\theta_{max}}\right)^2\right]$</td></tr>
<tr><td colspan="2">FENE potential between actin rings</td><td colspan="2">$U_{mt} = -\frac{1}{2} k_{mt}\, \Delta d_{max}\, ln\left[1 - \left(\frac{d - d_{eq}^{RR}}{\Delta d_{max}}\right)^2\right]$</td></tr>
<tr><th colspan="4">Parameters used in potentials</th></tr>
<tr><th colspan="2">Actin-Actin</th><th colspan="2">Actin-Spectrin</th></tr>
<tr><td rowspan="2">$U^{AA}(r)$</td><td>$K = 38\,\varepsilon/\sigma^2$</td><td rowspan="2">$U_{LJ}^{AS}(r)$</td><td>$E = \varepsilon$</td></tr>
<tr><td>$r_0 = 2^{1/6} \times 7\sigma$</td><td>$S = 4\sigma$</td></tr>
<tr><td rowspan="2">$U_{rep}^{AA}(r)$</td><td>$E = (98/3)\,\varepsilon$ (*)</td><th colspan="2" rowspan="2">Actin ring – Actin ring</th></tr>
<tr><td>$S = 7\sigma$</td></tr>
<tr><td rowspan="3">$U_{bending}$</td><td>$k_b = 3500\ K_B T$</td><td rowspan="3">U_{mt}</td><td>$k_{mt} = 19{,}822\ K_B T / d_{eq}^{RR}$</td></tr>
<tr><td>$\theta_0 \simeq 170.77^\circ$ (*)</td><td>$d_{eq}^{RR} = 185\, nm$</td></tr>
<tr><td>$\Delta\theta_{max} = 0.3\,\theta_0$ (*)</td><td>$\Delta d_{\max} = 0.3\, d_{eq}^{RR}$ (*)</td></tr>
<tr><th colspan="2">Spectrin-Spectrin</th><th colspan="2">Spectrin-Ankyrin</th></tr>
<tr><td rowspan="2">$U^{SS}(r)$</td><td>$K = 3.56\,\varepsilon/\sigma^2$</td><td rowspan="2">$U^{SK}(r)$</td><td>$K = 3.56\,\varepsilon/\sigma^2$</td></tr>
<tr><td>$r_0 = 2^{1/6}\sigma$</td><td>$r_0 = 2^{1/6} \times 3\sigma$</td></tr>
<tr><td rowspan="2">$U_{rep}^{SS}(r)$</td><td>$E = (1/16)\,\varepsilon$ (*)</td><td rowspan="2">$U_{rep}^{SK}(r)$</td><td>$E = (9/16)\,\varepsilon$ (*)</td></tr>
<tr><td>$S = \sigma$</td><td>$S = 3\sigma$</td></tr>
</table>

(*) Indicates that the parameter does not correspond to a well-defined measurable quantity but it rather represents an effective parameter that embodies a host of processes that are ignored here. All other parameters are defined based on experimental data.
