# Supplementary material for: Modeling of the axon membrane skeleton structure and implications for its mechanical properties
Source: PLoS Comput Biol. 2017 Feb 27;13(2):e1005407. doi: 10.1371/journal.pcbi.1005407 (PMC5348042; doi:10.1371/journal.pcbi.1005407)
Supplement: S2 Table — (PDF) [file pcbi.1005407.s019.pdf]

S2 Table. **Values of** $B\left(\times 10^{-3}\right)$ **for Young's modulus** E **ranging from** $1\sim 10\,kPa$ **and thicknesses** h **from** $5\sim 10\,nm$**.**

h (nm) \ E (kPa)	1	2	3	4	5	6	7	8	9	10
5	3.45	5.23	9.11	11.02	14.68	15.53	19.43	23.13	24.61	29.86
6	3.89	5.92	10.28	12.43	16.56	17.52	21.92	26.12	27.78	33.68
7	4.24	6.43	11.20	13.55	18.05	19.10	23.89	28.44	30.27	36.72
8	4.92	7.43	12.93	15.64	20.84	22.05	27.59	32.84	34.94	42.40
9	5.21	7.89	13.75	16.64	22.16	23.45	29.33	34.92	37.16	45.08
10	5.42	8.21	14.31	17.33	23.04	24.38	30.51	36.32	38.64	46.92
