# Supplementary material for: Modeling of the axon membrane skeleton structure and implications for its mechanical properties
Source: PLoS Comput Biol. 2017 Feb 27;13(2):e1005407. doi: 10.1371/journal.pcbi.1005407 (PMC5348042; doi:10.1371/journal.pcbi.1005407)
Supplement: S3 Table — (PDF) [file pcbi.1005407.s020.pdf]

**S3 Table**. **Values of $\beta$ for Young's modulus $E$ ranging from $1 \sim 10\, kPa$ and thicknesses $h$ from $5 \sim 10\, nm$.**

| *E (kPa)* / *h (nm)* | 1 | 2 | 3 | 4 | 5 | 6 | 7 | 8 | 9 | 10 |
|---|---|---|---|---|---|---|---|---|---|---|
| 5 | 1.3392 | 1.3429 | 1.3375 | 1.3395 | 1.3412 | 1.3387 | 1.3376 | 1.3392 | 1.3411 | 1.3396 |
| 6 | 1.3416 | 1.3428 | 1.3432 | 1.3415 | 1.3434 | 1.3421 | 1.3425 | 1.3436 | 1.3413 | 1.3425 |
| 7 | 1.3479 | 1.3482 | 1.3472 | 1.3479 | 1.3478 | 1.3482 | 1.3481 | 1.3456 | 1.3427 | 1.3483 |
| 8 | 1.3528 | 1.3521 | 1.3529 | 1.3518 | 1.3522 | 1.3516 | 1.3526 | 1.3572 | 1.3528 | 1.3564 |
| 9 | 1.3654 | 1.3652 | 1.3658 | 1.3656 | 1.3655 | 1.3652 | 1.3664 | 1.3687 | 1.3647 | 1.3683 |
| 10 | 1.3659 | 1.3742 | 1.3657 | 1.3643 | 1.3683 | 1.3715 | 1.3695 | 1.3658 | 1.3724 | 1.3762 |
